# Cancer inequalities in the United Kingdom and the data used to measure them: a scoping review

**DOI:** 10.1016/j.lanepe.2025.101246

**Published:** 2025-03-04

**Authors:** Sarah Underwood, Emre Oguzman, Judith Cole, Raphael Goldacre, Nileema Patel, David Davies, Claire Friedemann-Smith, F Lucy Wright, Ben Lacey, Brian D. Nicholson, Sasha Shepperd, Mark Lawler, Eva J.A. Morris

**Affiliations:** a Applied Health Research Unit, Big Data Institute, Nuffield Department of Population Health, University of Oxford, UK; b Cancer Theme, Nuffield Department of Primary Care Health Sciences, University of Oxford, UK; c Patrick G Johnston Centre for Cancer Research, Queens University Belfast, Belfast, UK; d DATA-CAN, The UK's Health Data Research Hub for Cancer, University of Leeds, UK

**Keywords:** Cancer, Inequality, Inequity

## Abstract

Significant cancer inequalities may exist across the United Kingdom (UK). Data are required to delineate and quantify these inequalities. This scoping review was undertaken to map the research evidence on UK cancer inequalities and determine the current data available, and the data gaps, that, if filled, could inform a strategy to reduce them. 444 studies were included. Their distribution across inequality domains, care pathways and cancer sites was uneven. The majority of studies were based on administrative datasets, notably cancer registry data, with a wide-range of methods used to define inequality groups. No UK-wide population-based evidence was identified. The landscape of data available in the UK to study cancer inequalities is uneven. Although there is a large volume of evidence available, there remain major gaps in both the data available and the knowledge base they are deployed to generate. This deficit needs to be addressed as a matter of urgency.

## Introduction

Although the National Health Service (NHS) seeks to ensure that everyone in the United Kingdom (UK) receives the best cancer treatment and attains the best outcomes, there is growing concern that there is persistent and significant variation in the quality of cancer care and outcomes across the country.^1^, ^2^, ^3^ Differences have been suggested to be wide-ranging and to span all aspects of the disease course and its management, from the risk of developing cancer, to the treatment received, and the outcome experienced.

In order to develop interventions to eliminate inequalities, it is essential to capture their occurrence, magnitude and frequency, investigate why they arise and understand what impact they have on patient outcomes. There have been numerous efforts to investigate and report trends, but as a result of differences in the quality and completeness of the data available across the UK, and a lack of co-ordination across analytical efforts, there remain many significant gaps in our knowledge base.^4^ There is additionally a lack of clarity on what data are available, or required, to map, monitor and investigate cancer inequalities across the UK.^5^

Recognising these gaps, a consortium of UK cancer charities commissioned this scoping review as part of efforts to develop a unified view of the data available, and required, to quantify cancer inequalities in the UK and their mitigation. As a scoping review, the aim is to describe the landscape of the existing literature on inequalities for cancer care and outcome in the UK, and the data which have been used, rather than to evaluate the findings of these studies. The resulting evidence is intended to help inform a strategy to more robustly quantify and understand cancer inequalities in the UK and so, ultimately, help reduce, and where possible, eliminate them.

There are two objectives for this scoping review. Firstly, to systematically map the available research evidence related to cancer inequalities in the UK. Secondly, to determine gaps in the availability of data to quantify and understand inequalities in cancer incidence, care and outcomes in the UK.

## Methods

The scoping review was undertaken to systematically explore the evidence available related to cancer inequalities (defined as a systematic difference between social groups) across the cancer continuum, from risk factors to treatment and outcome and the data used in these studies. The review is reported according to the Preferred Reporting Items for Systematic Reviews and Meta-Analyses extension for scoping reviews known as PRISMA-ScR.^6^

### Search strategy

A comprehensive literature search (using a broad range of search terms including all cancer types and inequalities (Appendix 1) was undertaken in Medline, CINHAL, PsychInfo and Embase and managed in Covidence, for peer-reviewed articles published in English from Jan 1, 2010 to Oct 1, 2023. The references of relevant review articles were searched.

### Study selection process

Inclusion and exclusion criteria for studies followed the population, concept and context categories for scoping reviews. The population inclusion criteria demanded that studies should involve individuals with cancer and report on ‘inequality’ across population groups. Inequality was defined as a difference across the population and between different groups. For concept, included studies were required to report on an aspect of cancer occurrence, diagnosis, treatment, care or outcome. Finally, the context of included studies was the UK population. Both qualitative and quantitative scientific papers were eligible to be included but opinion papers, editorials, conference abstracts, case reports and reviews were excluded.

Study selection criteria were first piloted with an initial review of a random sample of 25 titles and abstracts. Once a consensus on approach had been reached, all identified study abstracts were screened by two members of the reviewing team. This was done independently, with reviewers only discussing results once screening was complete. Disagreements were resolved by discussion with other members of the reviewing team.

A full-text review was then undertaken independently by two reviewers to identify potentially relevant studies. Studies where there was any uncertainty were reviewed by a third member of the team to determine eligibility.

### Extracting and charting the data

A data extraction form was piloted using a subset of studies and then deployed universally. Two members of the reviewing team extracted data using this form and disagreements were, again, resolved by a third reviewer. Extracted data related to study characteristics (year of publication, study population size, study period, country of study, study design and data sources), the type of cancer, the point on the care pathway the data related to and the inequality investigated. A summary of the main data items extracted, and their categorisation, are provided in the Panel 1.

Panel 1 **Selected data extraction categories and response options**

**Cancer type**

- Any cancer
- Bone/sarcoma
- Brain
- Breast
- Cancer – general (includes multiple types)
- Colorectal
- Gynecological
- Haematological
- Head & neck
- Liver
- Lung
- Oesophagogastric
- Other cancer
- Other gastrointestinal
- Pancreatic
- Prostate
- Urological (non-prostate)
- Skin

**Geographical area of the study**

- England
- International
- Northern Ireland
- Regional
- Scotland
- UK
- Wales

**Inequality studied**

- Age
- Comorbidity
- Socioeconomic status, education or employment
- Ethnicity
- Geography
- LGBTQ+
- Other inequality
- Sex

**Part of the care pathway studied**

- Access to care
- Awareness
- Diagnosis
- Experience
- Incidence
- Mortality
- Other area of care
- Prevalence
- Quality of life
- Risk
- Screening
- Survival
- Treatment

**Datasets used in the study**

- Audit
- Cancer Patient Experience Survey
- Civil registration
- Electronic Health Record
- Hospital discharge
- Other
- Primary care research
- Registry
- Screening
- Survey
- Trial
- Cancer waiting times

Study characteristics and findings were summarised narratively and organised by area of inequality. A web tool, structured around treemaps, that enables user interrogation of the studies by inequality area, cancer type and other study characteristics was created. To illustrate the frequency of studies by cancer type, area of the care pathway and overall, heatmaps were produced for each inequality area.

Stacked bar charts were used to show the frequency of the different data sources used in the studies identified by UK region as well as by inequality area. If a study used multiple (often linked) data sources then it was included in the count for each component dataset.

Information on the variables used to allocate individuals to relevant inequality groups was obtained during the data extraction process, alongside evidence of the strength and weaknesses of each approach. This information, alongside other evidence identified related to the quality of inequality data, is summarised in Table 1. No formal assessment of study quality was undertaken but the strengths and weaknesses of the evidence identified are presented narratively.

**Table 1:** The methods used to define inequality groups, their strengths and weaknesses and their frequency of use in the cancer inequality studies identified.

Inequality area	Group definition	Strengths	Weaknesses	Frequency of use		
				Number of studies using the method	%	Total number of studies
Age	Age at diagnosis, survey, death or admission	Available in majority of studies including at a population-level	Although information on chronological age is readily available, accompanying information on biological age, such as comorbidity or frailty levels, that may be more important than age when considering care options less available No consistent definition of ‘young’ or ‘old’ groups	103	100	103
Sex	Male and female	Available in the majority of studies including at a population-level	In the majority of studies it was unclear whether the information represented sex or gender	66	100	66
Socio-economic, education and employment	Ecological measures based on postcode mapping to lower super output areas and relevant socioeconomic deprivation indices.	Available in the majority of studies and at a population-level	Ecological measure so scores allocated to individuals may not truly represent their socioeconomic status Derivation of socioeconomic scores requires access to postcodes of residence which are a personal and sensitive data item so access strictly controlled and significant information governance challenges around appropriate use Numerous ecological measures used and no UK-wide measure preventing UK-wide analyses	228	88	258
	Education level, occupation and income level	Often captured from self-report so robust data Often captured at person level (although sometimes paternal occupation was used for studies of childhood cancers) so not an ecological measure	Education level, occupation and income level may not accurately represent socioeconomic status The measures used to allocated individuals to categories were not always clearly defined	73	28	258
Ethnicity	Derivation from Hospital Episodes Statistics or other routine dataset	Increasing capture of ethnicity data over time so ascertainment now over 90% and approaching population level	Depends on patient level linkage between datasets to determine ethnicity information. Patient-level linkage requires personal identifiers which are personal and sensitive data items so access strictly controlled and significant information governance challenges around appropriate use Multiple attendances at hospital can lead to multiple, and different, ethnicities for the same individual and uncertainty about which is the correct ethnicity to use Individuals may not identify with the standardised ethnicity groupings used	51	32	160
	Self-report	Provides the most robust information	Ethnicity reported may not fit into a standard ethnicity grouping	101	63	160
	Naming algorithm software such as OnoMap, Nam Pehchan or SANGRA	Some algorithms can provide allocation to detailed ethnic, cultural and linguistic groups	Requires access to the full names of all individuals in a study cohort. Names are a personal and sensitive data item so access strictly controlled and significant information governance challenges around appropriate use Some algorithms only identify one ethnicity grouping (for example South Asian or non-South Asian)	16	10	160
	Area-level or organisational-level ethnicity	Approach can enable population-based studies.	An ecological measure rather than a method that directly allocates individuals to an ethnic group	12	8	160
	Country of birth		Country of birth is not necessarily the same as ethnicity	4	3	160
Comorbidity	Summary comorbidity score, such as Charlson index	Possibly to calculate at a population-level from NHS datasets	Often depends on patient level linkage between datasets to determine comorbidity information. Person-level linkage requires personal identifiers which are personal and sensitive data items so access strictly controlled and significant information governance challenges around appropriate use The quality of the comorbidity score is dependent on the quality of the data it is generated from. This may reduce accuracy as, for example, scores derived from hospital discharge datasets will not include information on comorbidities managed in primary care but that may still be relevant to cancer care	25	43	58
	Presence or absence of a particular disease or condition	Enables detailed analyses of the impact of a specific, or a combination of diseases, on cancer care and outcome.	Often requires person level linkage to other datasets to identify presence or absence of the disease. Person-level linkage requires personal identifiers which are personal and sensitive data so access strictly controlled and significant information governance challenges around appropriate use The quality of the diagnostic information is linked to the quality of the data source used to derive it. This may reduce accuracy as, for example, information derived from hospital discharge datasets will not include information on comorbidities managed in primary care but that may still be relevant to cancer care Presence or absence of a disease alone does not provide any indication of the severity of a condition and well managed illnesses may have less impact on cancer care and outcome	25	43	58
	Receipt of a medicine or treatment used in the management of a disease	If prescribing information is available may enable study cohorts to be population-based	Receipt of a medicine does not necessarily indicate presence or absence of a health condition	2	3	58
	Self-report	Provides robust information	Challenging to capture information at a population level	3	5	58
	Number of days spent as an inpatient in hospital	Through linkage to hospital discharge datasets may enable study cohorts to be population-based	Number of days spent as an inpatient in hospital may not accurately represent level of comorbidity	1	2	58
	Other	Can provide more information about the severity of a particular condition	Broad range of definitions used (including anxiety or depression scales, fatigue and quality of life) making inter-study comparisons difficult	2	3	58
Sexual orientation and gender identify	Self-report of sexual history, orientation of gender identity	Provides robust information	Many people or participants may prefer not to reveal such sensitive information so studies not fully representative of the population	6	86	7
	Use of gender affirming hormone use/procedures	If prescribing information is available may enable study cohorts to be population-based	Use of hormones or procedures only provides surrogate information and may not be a true indication of a person's gender orientation	1	14	7
Geographical and provider	Travel time	Provides insight into the accessibility of care services	Calculation of travel time requires determination of the start (usually home) and end point of the journey but unclear which is the most appropriate points to use (for example to GP, secondary or tertiary hospitals) Calculation of travel time requires use of postcode which is a personal data item (as could enable patient identification) so access is strictly controlled and there are significant information governance challenges around appropriate use Travel time alone does not take into account other factors relevant to journey time such as access to a car or public transport	12	22	55
	Rural versus urban area of residence	Provides insight into the accessibility of care services	A simple dichotomy of rural and urban doesn't take into account the significant variation in rural or urban areas. For example, extremely remote rural areas and those with better access to services or inner city areas and market towns Allocation to rural/urban groups requires use of postcode which is a personal data item (as could enable patient identification) so access is strictly controlled and there are significant information governance challenges around appropriate use A rural and urban classification alone does not take into account other factors relevant to health care access such as access to a car or public transport	15	27	55
	NHS organisations and regions	Provides useful information to help directly inform care planning and services	NHS geographies change frequently preventing comparisons over time and, due to time lags in availability and access to data, may hinder timely and relevant reporting NHS organisations, such as hospitals, may offer care to very different populations. For example, some specialist centres may treat advanced cancers or high-risk patients. Comparisons between organisations need, therefore, to take into account this ‘casemix’ but detailed information on this may not be available (for example information on patient frailty or performance status and extent of disease)	21	38	55
	Geographic location or feature	Provides insight into the accessibility of care services	Broad range of definitions used for geographic location of characteristic (for example greenspace, city or region) making inter-study comparisons challenging	7	13	55

### Ethical approval and consent to participate

This study is a scoping review of published evidence and, as such, did not require research ethics committee approval or any mechanism to consent patient participation.

### Role of the funding source

The funders of this review had no role in study design, data collection, data analysis, data interpretation, writing the report or decision to submit.

## Results

**Fig. 1:**
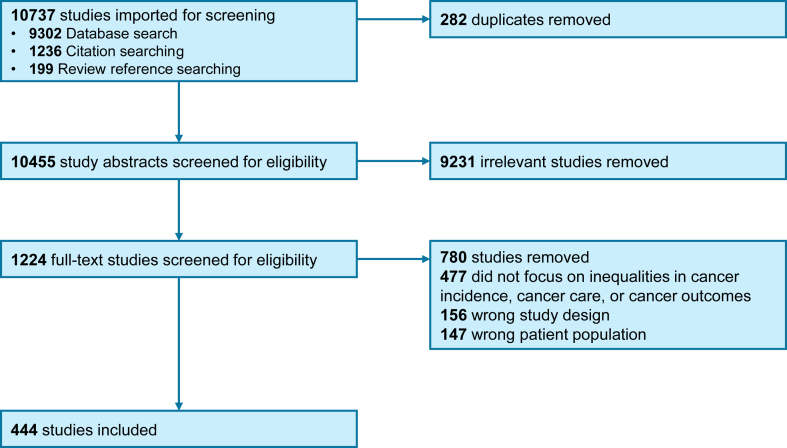
**PRISMA diagram illustrating the study identification and selection process**.

**Fig. 2:**
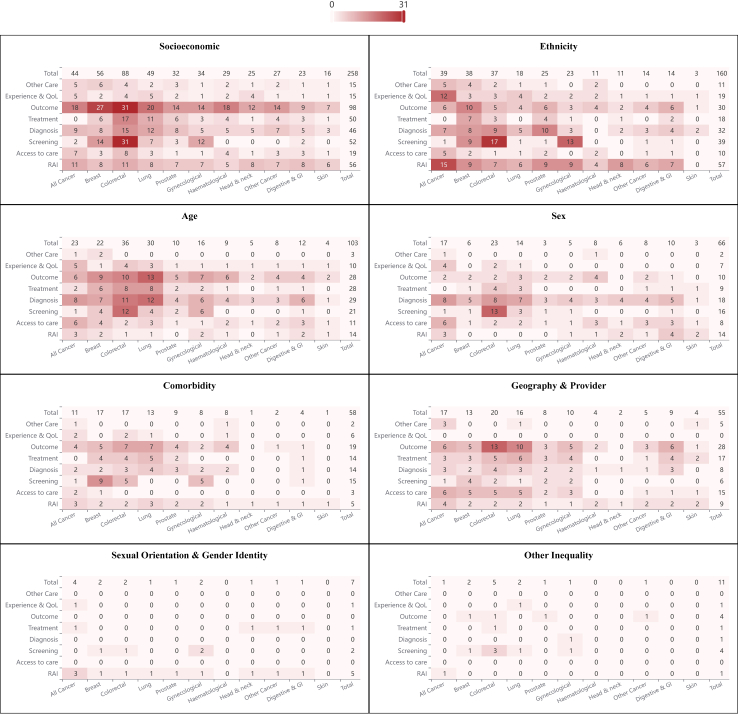
**Heatmaps showing the number of included studies by cancer type (x-axis) and area of the care pathway (y-axis) for each of the cancer inequality groups**. Abbreviations: RAI, Risk, awareness and incidence; QOL, quality of life.

The literature search, after de-duplication, identified a total of 10,455 citations (Fig. 1). Following screening the titles and abstracts of these studies, a total of 1224 went forward for full text review. Of these, 444 met the inclusion criteria and form the basis of this review. The majority of these studies focussed on the most common cancer types (colorectal (n = 128), breast (n = 109), lung (n = 76) and prostate (n = 56)), while the inequalities most frequently investigated related to either socioeconomic status (n = 258) or ethnicity (160). The number and frequency of studies for each cancer type by area of care is shown in Fig. 2. These studies were based on a range of data sources but cancer registry data were most frequently used, either alone or with linkage to another dataset, in just under half of the studies (n = 211). The majority of studies were undertaken within, or included data from, England (n = 340). Full details of all the studies, and a summary of their findings, are available through the on-line webtool at https://trainingidn.shinyapps.io/cancer_ineq_sr_app/.

### Socioeconomic status, education and employment-related inequalities

Over half (258, 58%) the studies identified investigated cancer inequalities in relation to socioeconomic status, income, education and employment. Their distribution across cancer type, and the aspect of care they examined, are illustrated in Fig. 2. Studies investigating socioeconomic inequalities with regard to risk, awareness and incidence, screening, diagnosis and outcome were the most common, particularly with a focus on colorectal cancer.

Across these studies, numerous different methods were used to determine and allocate individuals to different socioeconomic status, education and employment groups. In the population-based studies, the most common approach was assigning people to categories of an ecological measure of deprivation based on their postcode of residence. This was most commonly the English, Scottish or Welsh Index of Multiple Deprivation (IMD), followed by the Townsend and Carstairs scores and, more rarely, the use of the Mosaic and ACORN systems. Other measures used included highest educational level, employment grade, status or occupation, housing tenure, car ownership, economic activity, annual household income, proportion of income from benefits and, for studies looking at childhood cancers, paternal occupation.

### Ethnicity-related inequalities

Inequalities in relation to ethnicity were investigated in 160 studies. Fig. 2 illustrates their spread across cancer site and area of the care pathway. Breast cancer was the most common cancer site included in such studies, but there were reasonably large numbers across other common cancers and studies looking at multiple cancer sites.

Different methods were used to assign individuals to ethnic groups. The most common approach across the studies was to use the self-reported data held within the English Hospital Episode Statistics dataset or other smaller cohorts (n = 105). Naming algorithms (such as SANGRA, Nam Pechan and OnoMap) that assign ethnic groups based on the names of individuals in any cohort were used by 16 studies. Other methods included an ecological approach using area-based measures of population size by ethnic groups and a collection of data from hospital records (n = 12).

### Age-related inequalities

Age-related inequalities in care were investigated in a total of 103 studies. Studies examining age-related inequalities in diagnosis, screening, outcome and treatment across multiple cancer types and breast, colorectal and lung cancer was common with age at diagnosis the most frequent measure of age. There was no consistent approach, however, in the age thresholds used to define young or old populations.

### Sex-related inequalities

**Fig. 3:**
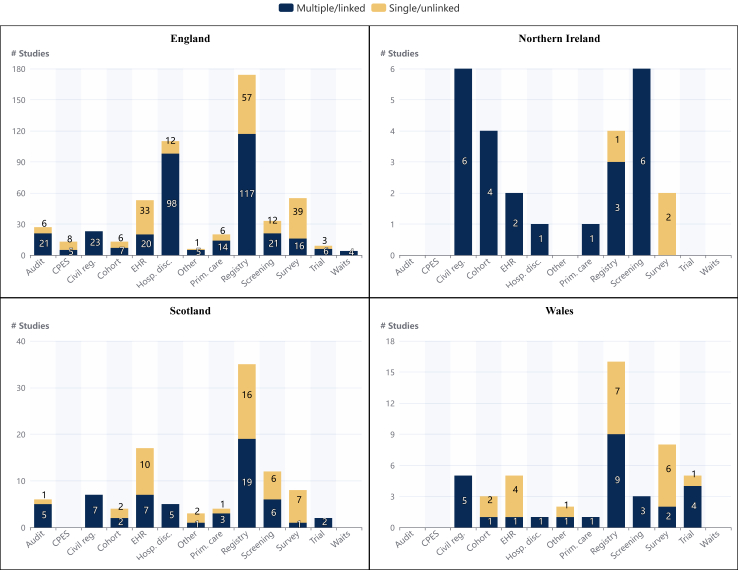
**The number of studies using the different data sources by country of the UK**. Data drawn directly from patient management systems was classed as Electronic Health Record (EHR) data. Data drawn from hospital systems with a primary purpose of administration was considered hospital discharge datasets (such as Hospital Episode Statistics, Scottish Morbidity Record 01 and Patient Episode Data Wales). Primary care research data was defined as that taken from a primary care research datasets such as Clinical Practice Research Datalink or QResearch). Nb: If a study uses multiple sources of data then it is included in the multiple/linked section of each relevant dataset category and so will be counted more than once. Abbreviations: CPES, Cancer Patient Experience Survey; EHR, Electronic Health Record; Hosp. Dis., Hospital Discharge; Prim. Care, Primary Care Research Data; Waits, Cancer Waiting Times.

Sixty-six studies investigated inequalities in relation to sex and their distribution across cancer types and by area of care is shown in Fig. 2. The data sources used by area of the UK are shown in Fig. 3. Sex was available in all these datasets deployed, although it was generally not clear whether the information provided related to biological sex or gender.

### Comorbidity-related inequalities

A total of 58 studies investigated inequalities in cancer care and outcome in relation to other diseases and conditions that individuals may possess. An equal number of these (n = 28) evaluated general health, predominantly measured using the Charlson comorbidity index, or looked at the presence of specific conditions. For example, 12 studies looked at the impact of mental health conditions and 2 on learning disabilities, predominantly focused on how they impacted on screening and diagnosis (Fig. 2). Other disease groupings including cardiovascular conditions, physical disabilities, or diabetes.

A range of methods were used to classify populations into presence or absence of a specific condition, including linkage to specialist disease registries, prescriptions for drugs specific to a condition or use of hospital discharge datasets to generate multimorbidity scores such as the Charlson comorbidity index. The majority of studies in this area were focused on breast, or other common, cancers and related to screening, diagnosis, treatment and outcome (Fig. 2).

### Geography- and provider-related inequalities

Fifty-five studies looked at geographical inequalities in care. Their spread by cancer type and area of the care pathway is show in Fig. 2. Studies looking at geographical variation in screening uptake and outcome were the most common, with the majority of studies focusing on breast, colorectal, lung or multiple cancer types.

Individuals within the studies were grouped into categories using a number of different methods. A total of 15 of the studies compared outcomes by living in rural or urban areas, 12 looked across different categories of travel time to hospital but the majority (28) compared outcomes across different NHS areas (for example, across NHS regions, cancer alliances, hospitals or multidisciplinary teams) or other geographies.

### Sexual orientation and gender identity-related inequalities

Only seven studies investigated inequalities related to sexual orientation and gender identity. The majority of these included more than one cancer, with only one focusing on a single type (cervical cancer) (Fig. 2). Sexual orientation and gender identity were not captured routinely in any of the population-based datasets commonly used, so the majority of the identified studies captured self-reported information within cohort studies on either LGBTQ + identity or self-reported sexual behaviour (e.g. same-sex sexual history). Only one study used a different approach of identifying gender non-confirming people through their use of gender-affirming hormone-treatment and procedures.

### Other inequalities

Eleven studies sought to investigate inequalities that do not fall into any of the previous categories. These covered diverse topics such as screening uptake in relation to religion and the specialisation and/or caseload of managing clinicians (Fig. 2).

### Data sources

**Fig. 4:**
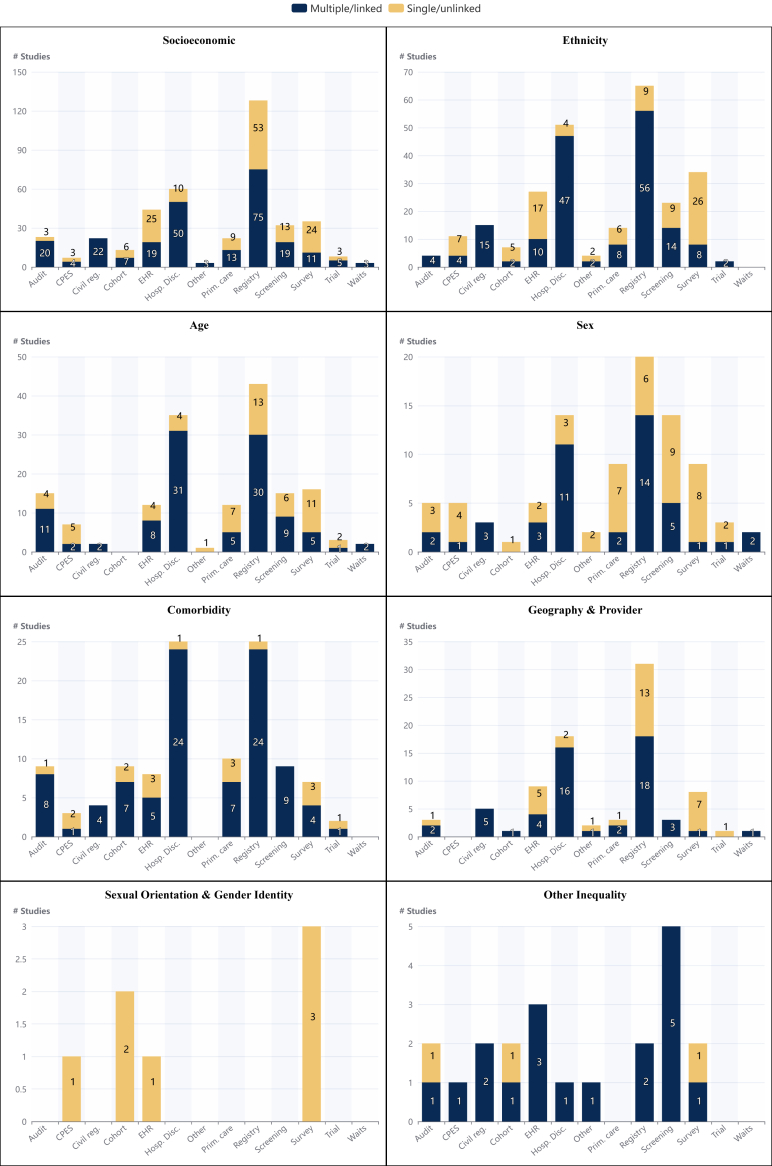
**The number of studies using the different data sources by cancer inequality group**. Data drawn directly from patient management systems was classed as Electronic Health Record (EHR) data. Data drawn from hospital systems with a primary purpose of administration was considered hospital discharge datasets (such as Hospital Episode Statistics, Scottish Morbidity Record 01 and Patient Episode Data Wales). Primary care research data was defined as that taken from a primary care research dataset such as Clinical Practice Research Datalink or QResearch). Nb: If a study uses multiple sources of data then it is included in the multiple/linked section of each relevant dataset category and so will be counted more than once. Abbreviations: CPES, Cancer Patient Experience Survey; EHR, Electronic Health Record; Hosp. Dis., Hospital Discharge, Prim; Care, Primary Care Research Data; Waits, Cancer Waiting Times.

Figs. 3 and 4 illustrate the data sources used in the studies originating from each nation in the UK and deployed to investigate each inequality area respectively. Across all nations and groups, cancer registry data were the most frequently used data source and the majority of studies were centred on the use of administrative ‘routine’ datasets rather than bespoke research datasets.

### Quality of cancer inequality data

Table 1 summarises the methods used to allocate individuals to inequality groups, their frequency and the strengths and weaknesses of the different approaches. A wide range of methods were used and there were benefits and limitations for all of them. There were also some more general challenges identified, summarised in Table 2, including ensuring study cohorts are representative of the wider population and data availability, accessibility and appropriateness as well as development of methods for robustly investigating cancer inequalities.

**Table 2:** Evidence identified relevant to methods and data availability, accessibility and quality across all cancer inequality studies.

Area	Evidence
Representativeness	Individuals participating in inequality studies may not be representative of the general population
Data availability and accessibility	There may be a large number of potentially marginalised groups in society but little data available via which members can be identified. This may be because the information needed to identify members of relevant groups is likely to be highly sensitive so it is often not captured due to concerns about its appropriate use. Inequality data are often extremely sensitive information so, where it does exist, access is strictly controlled and there are significant information governance challenges around appropriate use No UK-wide studies undertaken. This is possibly due to challenges of accessing and bringing together data from England, Scotland, Wales and Northern Ireland Very few studies were identified which used full pathway data spanning, for example, risk, screening, primary, secondary and tertiary care. This is possibly due to challenges of accessing and bringing together relevant data assets captured, maintained and ‘controlled’ by different organisations.
Data quality and richness	Many of the administrative datasets used to study cancer inequalities were not originally intended to support research and so their appropriate use can be methodological challenging. Research to investigate data quality and inform their robust is extremely valuable. Many inequality indicators available at a population-level, or information on casemix, are relatively blunt. For example, indicators of socioeconomic status are most commonly ecological measures and measures of comorbidity and frailty, whilst predictive of outcome, have relatively low sensitivity and specificity. Again, research to enhance and improve indicators would be valuable

## Discussion

This study has identified a large body of research evidence investigating cancer inequalities across the UK. Studies investigating inequalities in socioeconomic status were most common, followed by ethnicity but there was considerably less evidence related to other potential inequality groups. The data these studies are based on are wide ranging. The majority were population-based studies, most frequently conducted using cancer registry and other administrative health datasets, but cohort and other study designs were also common. The extent of the evidence found also varied substantially by inequality area, aspect of the care pathway and the cancer type studied. This means that the cancer inequality evidence base landscape is, effectively, uneven (Fig. 2).

This imbalanced distribution of evidence is likely to be driven by both the existence, and accessibility, of the underpinning data. By their nature, the information required to allocate individuals to potentially marginalised groups in society is highly sensitive. This means that for many groups, the data that could identify members are not routinely captured or recorded, and when they are, access is strictly controlled. This was clear in the evidence based around cancer inequalities in the LGBTQ + community, with only seven studies being identified and all of these inferring sexual orientation from gender identity rather than having it clearly defined. There are, undoubtedly, many other minority groups (such as migrant groups or the homeless) for whom such identifying information is absent or inaccessible and without these data it will never be possible to robustly measure any inequalities, let alone generate evidence to help address them. If it is a priority to tackle cancer inequalities (which we believe it is), then efforts are required to consult with relevant communities to agree policy on acceptable approaches to capture, manage, and use such sensitive data for the common good.

Another closely related challenge is the depth of information available across inequality groups. For example, studies looking at inequalities related to area of residence such as socioeconomic status, travel time to hospital and differences between rural or urban area of residence all require the use of postcode of residence. With an average of only 15 houses in a postcode area, access to these data could enable individuals to be identified and so it is considered a highly sensitive variable and, for privacy reasons, access needs to be strictly controlled. This has led to many measures being grouped to larger areas and for socioeconomic inequalities this is often lower super output area. With these containing between 400 and 1200 households, and a usually resident population of between 1000 and 3000 people, it is highly likely that their characteristics will vary and allocations may not truly represent any individual's socioeconomic status. This, in turn, will reduce the accuracy of analyses undertaken. New approaches, such as use of the unique property reference number (UPRN),^7^^,^^8^ or enabling linkage to census data, would overcome this ‘ecological fallacy’^9^ and their more routine adoption could provide a new dimension in our understanding of what drives socioeconomic inequalities. However, their benefit for public good would need to be balanced against the need to protect privacy.^10^

This need to categorise individuals into broad population groups is relevant, but also a potential limitation, for all cancer inequality studies.^11^ For example, what is the cut-off to define old and young or rural and urban? What is the most appropriate distance over which to calculate travel time from home to GP, local or specialist hospital? Which measure of socioeconomic status is most representative and what ethnicity groups are more appropriate to use? Many different indicators and approaches were deployed across the studies identified, making inter-study comparisons challenging and contributing to the absence of UK-wide information on any inequality area.

A final data quality challenge was the depth and richness of the data available. For example, although chronological age was readily available, other factors closely associated with age and that could contribute to variation in care and outcome, such as frailty or comorbidity, were less accessible or robust. This is important, as many aspects of cancer care and outcome vary in relation to physical fitness and patient preference, so simply investigating variation in relation to chronological age may not yield readily actionable evidence. Similarly, this lack of detail on factors such as frailty and comorbidity was relevant to studies looking at variation across hospital providers, with different NHS organisations treating different patient populations. Failure to consider this patient ‘casemix’ may limit the validity of any comparisons made. Richer data containing information on frailty, patient choice and other such important factors would, therefore, enable more robust studies to be performed. This could be achieved by enabling linkage to primary care data or simply increasing investment in data quality and capture at provider level.

There are calls for a national strategy to optimise cancer services and outcomes, but this will only be effective if the evidence it is based on is strong and robust.^10^^,^^12^ High quality data are fundamental to generating such evidence and so a national framework and guidance for capturing such data to ensure sufficient granularity and robust coding would be highly advantageous and an essential component of such a national strategy.

With such a large number of studies identified through this scoping review, it is apparent there is a significant volume of research evidence around cancer inequalities in the UK. None of these studies, however, were able to use a UK-wide population-based dataset and so provide a truly national perspective. Although for some inequalities such studies are impossible, as the data simply do not exist, for others, such as age and sex, they are available and could be generated. Given a UK-wide perspective would be hugely valuable in informing allocation of resource and efforts to tackle inequalities the absence of such studies is important. Data access processes in each of the UK nations are cumbersome, lengthy and resource-intensive, so the absence of such studies is potentially a result of these challenges. Efforts to both simplify data access as well as to generate indicators that can be deployed across the UK (for example a UK-wide metric of socioeconomic status) would, again, enrich the evidence base and better inform interventions that can help tackle inequalities. Notably, several countries have already transformed the cancer outcomes they attain by creating and using national-level linked datasets to support evidence-based health policy decisions.^10^^,^^13^^,^^14^ In addition, whilst no large-scale dataset will ever be perfect, an in-depth understanding of the quality of the data it holds alongside the development of robust methods to analyse them will always strengthen their use. Several studies identified in his review sought to develop such methods and expansion of this area of work would be beneficial.

Given UK health data are some of the best in the world, if they can be harnessed effectively, the country is in a uniquely strong position to use them to transform our cancer services and drive improvements in care and outcome.

There was also significant duplication observed within the themes of the papers included in this scoping review. For example, 52 studies examined socioeconomic inequalities in relation to cancer screening. Despite the use of varying datasets and populations, these studies consistently show lower screening uptake in those individuals from more socioeconomically deprived areas, suggesting that the link is confirmed and further studies in this area are not required. Instead, research is needed to help understand what aspects of socioeconomic status explain this lower uptake and what can be done about it.

At present, the funding system demands academic teams compete for scarce funding to deliver research projects and these projects are perhaps driven by what data are available and what is readily achievable rather than what evidence is required. Although not investigated in this review, there may also be inequalities in data access, with some academic or health service groups having easier access than others. Perhaps a better co-ordinated approach to commissioning research would provide a more cost-effective solution to ensuring evidence gaps are filled and policy makers are provided with the information they need whilst also helping to reduce this research duplication.

The study team sought to undertake a comprehensive review of all peer-reviewed research examining cancer inequalities in the UK and although gold standard approaches have been used, this scoping review has a number of limitations. Firstly, our search strategy was designed to identify studies that overtly looked at inequalities, but this may not have identified all relevant data. This is because studies will, undoubtedly, have been published over the relevant period that may use potentially relevant categories (such as age, sex, socioeconomic status or ethnicity) in analyses as confounders. Such analyses may yield potentially valuable information on variation in care and outcome across our population, but if they have not overtly discussed inequalities or inequities, our search strategy may have failed to identify them.

Furthermore, the search strategy deployed focused on inequality-related groups based on individual characteristics and, consequently, this failed to return complete information on studies investigating provider-related variation. Given that variation in care (i.e. a ‘postcode lottery’) is a major concern, with a growing body of evidence suggesting significant variation in the quality of cancer services across the UK, this is a major limitation. The search strategy used, however, identified over 10,000 studies, including many that looked at provider variation, and given the available resources for the project, it was not feasible to revise the strategy. Capturing this evidence would, however, be extremely valuable and could be the subject of future studies, or an expansion and refresh of this current project.

Additionally, this review focuses on evidence published in peer-reviewed journals but, as cancer inequalities are highly relevant to the NHS and policy makers, there will undoubtedly be substantial grey literature, dashboards and other relevant tools that may have been omitted. Notably, this review did not include the hugely valuable National Cancer Audit Reports which focus on investigating variation in care across the NHS.^15^ Furthermore, whilst this review focused solely on cancer, any inequalities identified are likely to occur for other health conditions too. Relevant evidence may again, therefore, have been overlooked. Finally, because of the time frames of research publication, the review will not have identified work that may be currently underway. This may be significant, given the current level of concern about cancer inequalities in the UK. Ongoing monitoring of the data and evidence landscape would help ensure efficient and maximal use of the information available to help tackle the inequalities that exist.

This review has identified that there is a substantial body of evidence quantifying cancer inequalities across the NHS, but understanding how to use the evidence to reduce these inequalities is complex. Ten years ago, the largest evaluation of cancer inequalities in Europe at the time^3^ provided the evidence base to develop a European Cancer Patient's Bill of Rights^16^^,^^17^ which was adopted widely and had Europe wide impact. A potential focus of future investment could, therefore, be implementation and improvement science, so rather than simply describe inequalities across society we will have the more complete evidence base needed to tackle them robustly.

Inequalities in care and outcome are a major concern to the UK's cancer community and a priority to address as a matter of urgency. To achieve this, evidence that quantifies their size and what drives them is important and that demands detailed, relevant and robust data. Despite the large volume of evidence available in the UK, there remain major gaps in both the data available and the knowledge base they are used to generate. Until these gaps in both data and analysis are filled, it will be challenging to have evidence-based policies and interventions that can minimise the negative impacts of inequalities in cancer care in the UK. A co-ordinated effort from all stakeholders is, therefore, required to ensure appropriate data are gathered, managed, linked, made accessible and analysed effectively to understand and reduce cancer inequalities. Only then will NHS data be used efficiently and effectively to help improve care and outcome across the UK. Data, when used effectively, can save lives.

## Contributors

EJAM and ML conceived and designed the work. SU, EO, JC, RG, NP, DD, CFS, FLW and EJAM acquired and reviewed the papers and extracted data from them. SU, EO, JC, RG and EJAM synthesised the results with EO leading on data tools and visualisations. All authors contributed to the manuscript, approved the final version and the decision to submit, and agreed to be accountable for all aspects of the work.

## Data sharing statement

The dataset generated and/or analysed during the current study is available from the webtool located at https://trainingidn.shinyapps.io/cancer_ineq_sr_app/.

## Declaration of interests

Ben Lacey declares salary funding support from UK Biobank (funded largely by the Medical Research Council and the Wellcome Trust. Nileema Patel declares salary funding support from the Thames Valley Deanery and Oxford University Hospitals NHS Trust and payment/honoraria for teaching at Imperial College London. Brian D Nicholson declares salary funding support and holding grants from the National Institute of Health Research, Cancer Research UK and GRAIL. Mark Lawler declares honoraria from Pfizer for presentations unrelated to this work. All other authors declare no competing interests related to this work.
