# Supplementary material for: Cancer inequalities in the United Kingdom and the data used to measure them: a scoping review
Source: Lancet Reg Health Eur. 2025 Mar 4;52:101246. doi: 10.1016/j.lanepe.2025.101246 (PMC11925185; doi:10.1016/j.lanepe.2025.101246)
Supplement: Appendix [file mmc1.docx]

**Supplementary Appendix**

Underwood S, Oguzman E, Cole J, Goldacre R, Patel N, Davies D, Friedmann-Smith C, Wright FL, Lacey B, Nicholson BD, Shepperd S, Lawler M, Morris EJA. Cancer inequalities in the United Kingdom and the data used to measure them: a scoping review

**Appendix 1 – Search strategy for the scoping review entitled: Cancer inequalities in the UK and the data used to measure them: a scoping review**

| 1 | exp Neoplasms/ |
| --- | --- |
| 2 | (neoplas* or tumor* or tumour* or cancer* or malignan* or carcino* or sarcom* or leukaem* or leukem* or lymphom* or melano* or metastas* or mesothelio* or mesotelio* or carcinomatos* or gliom* or glioblastom* or osteosarcom* or blastom* or neuroblastom* or oncolog* or myelodysplas* or adenocarcinoma* or choriocarcinoma* or myeloma*).ti. |
| 3 | or/1-2 |
| 4 | exp Health Services Accessibility/ |
| 5 | Healthcare Disparities/ |
| 6 | exp health inequities/ |
| 7 | prejudice/ or ageism/ or gender equity/ or homophobia/ or exp racism/ or sexism/ |
| 8 | Ethnic and Racial Minorities/ |
| 9 | racial groups/ or exp blacks/ or exp asians/ or ethnicity/ |
| 10 | exp "emigrants and immigrants"/ or refugees/ |
| 11 | Minority Groups/ |
| 12 | exp Socioeconomic Factors/ |
| 13 | vulnerable populations/ |
| 14 | disabled persons/ or persons with mental disabilities/ or learning disabilities/ or exp intellectual disability/ or exp Autism Spectrum Disorder/ |
| 15 | bisexuality/ or exp homosexuality/ or transsexualism/ or exp "sexual and gender minorities"/ |
| 16 | sex work/ or sex workers/ |
| 17 | criminals/ or prisoners/ |
| 18 | enslaved persons/ |
| 19 | alcoholics/ or drug users/ |
| 20 | exp homeless persons/ or working poor/ |
| 21 | (prejudice or racism or racist or sexism or sexist or ageism or ageist or homophobi? or transphobi? or xenophobi? or ((age? or gender* or sex*) adj3 discriminat*)).ti,ab,kf. |
| 22 | (ethnic* or race or racial or religious or religion? or islam* or hindu* or sikh* or judaism).ti,ab,kf. |
| 23 | (blacks or (black adj2 (british or people or person? or population? or men or male? or women or female?)) or afrocaribbean or african? or caribbean or asian? or pakistani? or indian? or bangladeshi? or bengali? or punjabi or sri lankan? or nepali? or nepalese or afghani? or chinese? or jew? or jewish or muslim? or punjabi? or bengali?).ti,ab,kf. |
| 24 | (traveller? or roma? or gypsy or gypsies).ti,ab,kf. |
| 25 | (migrant? or immigrant? or refugee? or asylum seeker?).ti,ab,kf. |
| 26 | (lgb* or homosexual* or gay? or lesbian? or bisexual* or "men who have sex with men" or "women who have sex with women" or sexual orientation or transgender* or transexual* or trans men or trans male? or trans women or trans female? or nonbinary or non-binary or gender fluid* or gender identit*).ti,ab,kf. |
| 27 | (((vulnerable or disadvantage* or poor) adj2 (people or person? or population? or communit*)) or (impoverish* or poverty or disparity or disparities)).ti,ab,kf. |
| 28 | (inequalit* or inequit* or equality or equity or disparit*).ti. or ((health or socioeconomic or social or economic) adj2 (inequalit* or inequit* or equality or equity or disparit*)).ti,ab,kf. |
| 29 | ((disabled or disability) adj2 (people or person? or population?)).ti,ab,kf. |
| 30 | (((intellectual or learning) adj2 (disabilit* or difficult*)) or (down* syndrome or asperger* or autism or autistic or global developmental delay* or william* syndrome or fragile x or cerebral palsy)).ti,ab,kf. |
| 31 | ((education* or academic) adj2 (status or attainment or achievement)).ti,ab,kf. |
| 32 | (homeless* or (housing adj2 (poor or condition? or stab* or instab* or unstab*))).ti,ab,kf. |
| 33 | (sex work* or prostitut*).ti,ab,kf. |
| 34 | (crime or criminal? or prison* or incarcerat*).ti,ab,kf. |
| 35 | ((alcohol or drug? or substance) adj2 (abuse or dependen* or addict*)).ti,ab,kf. |
| 36 | (lonely or loneliness or (social* adj2 (isolat* or exclu*))).ti,ab,kf. |
| 37 | (modern slave* or (enslav* adj2 (worker? or people or person? or population?))).ti,ab,kf. |
| 38 | or/4-37 |
| 39 | exp United Kingdom/ |
| 40 | (national health service* or nhs*).ti,ab,in. |
| 41 | (english not ((published or publication* or translat* or written or language* or speak* or literature or citation*) adj5 english)).ti,ab. |
| 42 | (gb or "g.b." or britain* or (british* not "british columbia") or uk or "u.k." or united kingdom* or (england* not "new england") or northern ireland* or northern irish* or scotland* or scottish* or ((wales or "south wales") not "new south wales") or welsh*).ti,ab,jw,in. |
| 43 | (bangor or "bangor's" or cardiff or "cardiff's" or newport or "newport's " or st asaph or "st asaph's" or st davids or swansea or "swansea's").ti,ab,in. |
| 44 | (aberdeen or "aberdeen's" or dundee or "dundee's" or edinburgh or "edinburgh's" or glasgow or "glasgow's" or inverness or (perth not australia*) or ("perth's" not australia*) or stirling or "stirling's").ti,ab,in. |
| 45 | (armagh or "armagh's" or belfast or "belfast's" or lisburn or "lisburn's" or londonderry or "londonderry's" or derry or "derry's" or newry or "newry's").ti,ab,in. |

**Supplementary Table 1: A summary of the studies included in the scoping review and the inequality area they investigated.**

Further information, and the ability to interrogate the studies in more detail (for example identify all studies relevant to a particular cancer type or area of care) is available in the online tool accessible at <https://trainingidn.shinyapps.io/cancer_ineq_sr_app/>

Studies in italics sought to investigate data quality or develop, or strengthen, methods to enable more robust analysis

| First author | Paper Title | Summary of Findings | Journal | Year of publication | Inequality area studied | | | | | | | |
| --- | --- | --- | --- | --- | --- | --- | --- | --- | --- | --- | --- | --- |
|  |  |  |  |  | Age | Comorbidity | Socioeconomic status, education and employment | Ethnicity | Geography | LGBTQ+ | Other Inequality | Sex |
| *Abdel-Rahman* | *No socioeconomic inequalities in ovarian cancer survival within two randomised clinical trials* | *In a clinical trial setting there is no socioeconomic deprivation gradient in survival suggesting the differences observed in the general population are due to inequalities in care* | *British Journal of Cancer* | *2014* |  |  | *X* |  |  |  |  |  |
| Abel | Emergency diagnosis of cancer and previous general practice consultations: insights from linked patient survey data | Greater risk of no prior consultations before emergency presentation in people living in people in the lowest SES group, as well as in older people and males. Effects were greatest for those with oral/oropharyngeal, anal, laryngeal and small intestine cancers | British Journal of General Practice | 2017 | X |  | X |  |  |  |  | X |
| *Abel* | *Post-sampling mortality and non-response patterns in the English Cancer Patient Experience Survey: Implications for epidemiological studies based on surveys of cancer patients* | *Older age associated with post sampling mortality. Survey response rate lower in non-white and affluent patients* | *Cancer Epidemiology* | *2016* | *X* |  | *X* | *X* |  |  |  |  |
| Abel | Cancer-specific variation in emergency presentation by sex, age and deprivation across 27 common and rarer cancers | Younger people with acute leukaemia, colon, stomach and oesophageal cancer were at a higher risk for emergency presentation. Older patients with laryngeal, thyroid and oral cancer and melanoma and Hodgkin’s lymphoma were at higher risk of emergency presentation. Females at greater risk of emergency presentation than males for bladder, brain, liver, stomach, colon and lung cancer. Males were at greater risk of presentation than females for oral/oropharyngeal cancer, lymphomas and melanoma. Around a third of people presenting as an emergency from lower SES groups had no prior consultation with a GP indicative of a ‘missed’ diagnosis. | British Journal of Cancer | 2015 | X |  | X |  |  |  |  | X |
| Abou-Chedid | The impact of socio-economic deprivation on recovery following robotic assisted radical cystectomy | Recovery was independent of socioeconomic status | Urologia | 2023 |  |  | X |  |  |  |  |  |
| Adam | Do patients' faces influence General Practitioners' cancer suspicions? A test of automatic processing of sociodemographic information | Gender influenced the likelihood of GP suspicion of cancer but this was influenced by age. GP suspicion of cancer risk was modified by ethnicity: e.g. more likely to suspect cancer in young Caucasian makes than young Asian males | PloS One | 2017 | X |  |  | X |  |  |  | X |
| Affar | Cervical cancer incidence by ethnic group in Scotland from 2008 to 2017: A population-based study | Lower incidence of cervical cancer in ethnic minorities compared to the White population | European Journal of Cancer Care | 2021 |  |  |  | X |  |  |  |  |
| Afshar | Do Learning Disabilities Affect Testicular Cancer Survival: A National Cohort Study Between 2001 and 2015 | People with learning disabilities are less likely to have their cancer diagnosed at an early stage and so have worse survival outcomes | European Urology Oncology | 2020 |  | X |  |  |  |  |  |  |
| Aitken | Mammographic density and markers of socioeconomic status: a cross-sectional study | SES gradients in percent density parallels known SES gradients in breast cancer risk | BMC Cancer | 2010 |  |  | X |  |  |  |  |  |
| *Alessy* | *How representative are colorectal, lung, breast and prostate cancer patients responding to the National Cancer Patient Experience Survey (CPES) of the cancer registry population in England? A population-based case control study* | *Respondents are more likely to be white than non-respondents. Respondents ate more likely to be affluent than non-respondents. Median survival of responders greater than non-responders* | *BMJ Open* | *2019* |  |  | *X* | *X* |  |  |  |  |
| Ali | Anal cancer survival: a socioeconomic analysis | No relationship between SES and anal cancer survival | Annals of the Royal College of Surgeons of England | 2021 |  |  | X |  |  |  |  |  |
| Ali | Incidence of gastrointestinal cancers by ethnic group in England, 2001-2007 | Non-white groups had lower incidence of colorectal, oesophageal and pancreatic cancer and higher incidence of liver and gallbladder cancer compared to white groups. Gastric cancer incidence was higher in south Asian, but lower in Black and Chinese groups. The risk of gastrointestinal cancer varies by ethnic group including within groups that are often grouped together (e.g. south Asian and black patients) | Gut | 2013 |  |  |  | X |  |  |  |  |
| Ali | Cancer incidence in British Indians and British whites in Leicester, 2001-2006 | Cancer incidence in British Indians is lower than in British Whites, particularly for cancer of the breast, prostate, colon and lung, but much higher than for the same ethic group in India | British Journal of Cancer | 2010 |  |  |  | X |  |  |  |  |
| Ali | Barriers to uptake among high-risk individuals declining participation in lung cancer screening: a mixed methods analysis of the UK Lung Cancer Screening (UKLS) trial | Non-uptake is associated with female gender, older age, and lower socioeconomic group | BMJ Open | 2015 | X |  | X |  |  |  |  | X |
| Allen | Is social inequality related to different patient concerns in routine oral cancer follow-up clinics? | No difference in patient concerns by SES | European Archives of Oto-Rhino-Laryngology : | 2017 |  |  | X |  |  |  |  |  |
| Amin | Osteonecrosis in patients with acute lymphoblastic leukaemia: a national questionnaire study | Asian patients have a higher risk of osteonecrosis than White patients | BMJ Paediatrics Open | 2017 |  |  |  | X |  |  |  |  |
| Amr | Variation in survival after surgery for peri-ampullary cancer in a regional cancer network | Although there is variation in the provision and outcome of surgery for PC between regional hospitals, this is not adversely affected by geographical isolation from the regional centre. | BMC Surgery | 2017 |  |  |  |  | X |  |  |  |
| Arik | Socioeconomic disparities in cancer incidence and mortality in England and the impact of age-at-diagnosis on cancer mortality | Considerable disparities in cancer incidence and mortality in relation to SES. There is a significant increase in cancer deaths due to delays in pathways | PloS One | 2021 |  |  | X |  |  |  |  |  |
| Askari | Colorectal tumour characteristics and oncological outcome in patients with inflammatory bowel disease | IBD was not a predictor of disease-free survival. Patients with IBD had higher rates of stoma formation, longer hospital stays, and were younger | Surgical Practice | 2020 |  | X |  |  |  |  |  |  |
| Askari | Who requires emergency surgery for colorectal cancer and can national screening programmes reduce this need? | Black/Caribbean colorectal cancer patients were more likely to undergo emergency surgery, placing them at higher risk of poor outcome. People with lower SES are more likely to present, and require surgery, as an emergency | International Journal of Surgery | 2017 | X |  | X | X |  |  |  | X |
| Askari | The relationship between ethnicity, social deprivation and late presentation of colorectal cancer | Black Afro/Caribbean patients are diagnosed with colorectal cancer at a younger age than White-British patients, and are more likely to present with stage IV tumours. People with lower SES are more likely to present with advanced tumours | Cancer Epidemiology | 2017 |  |  | X | X |  |  |  |  |
| Atherton | Differences in self-assessed health by socioeconomic group amongst people with and without a history of cancer: an analysis using representative data from Scotland | Self assessed health amongst people with a history of cancer was more likely to be poor in socioeconomically deprived groups | Journal of Cancer Survivorship | 2012 |  |  | X |  |  |  |  |  |
| Bailey | Sociodemographic variations in the uptake of faecal immunochemical tests in primary care: a retrospective study | People aged 65 years or older were more likely to return a faecal immunochemical test (FIT). People from ethnic minorities less likely to return a FIT compared to White people. Males less likely to return a FIT. Unreturned FIT more than doubled in most deprived quintile compared to least deprived | British Journal of General Practice | 2023 | X |  | X | X |  |  |  | X |
| Bailey | Socioeconomic indicators of health inequalities and female mortality: a nested cohort study within the United Kingdom Collaborative Trial of Ovarian Cancer Screening (UKCTOCS) | Women living in more deprived areas continue to have higher ovarian cancer mortality | BMC Public Health | 2015 |  |  | X |  |  |  |  |  |
| Baker | p53 mutation, deprivation and poor prognosis in primary breast cancer | p53 mutation is more common in women with breast cancer residing in more socioeconomically deprived areas | British Journal of Cancer | 2010 |  |  | X |  |  |  |  |  |
| Balata | Attendees of Manchester's Lung Health Check pilot express a preference for community-based lung cancer screening | Mobile CTs canners were preferred for community based lung cancer screening in the most deprived groups | Thorax | 2019 |  |  | X |  |  |  |  |  |
| Bang | Primary care factors associated with cervical screening coverage in England | Cervical screening coverage is lower in practices serving higher proportions of ethnic minority patients. Cervical screening uptake was negatively associated with socioeconomic status | Journal of Public Health | 2012 |  |  | X | X |  |  |  |  |
| Banks | Preferences for cancer investigation: a vignette-based study of primary-care attendees | People aged 60-69 were more likely to opt for testing than people 40-49 or 70+. More affluent patients attending primary care had a stronger preference for requesting diagnostic tests for colorectal and pancreatic cancer symptoms. Those with shorter travel times to the testing centre expressed a preference for diagnostic tests for colorectal and lung cancer | The Lancet Oncology | 2014 | X |  | X |  | X |  |  |  |
| Bansal | Major ethnic group differences in breast cancer screening uptake in Scotland are not extinguished by adjustment for indices of geographical residence, area deprivation, long-term illness and education | Compared with White and Chinese groups, Pakistani, African, Indian, and other South Asian groups are less likely to attend breast cancer screening | British Journal of Cancer | 2012 |  |  |  | X |  |  |  |  |
| Barbiere | The association of diagnosis in the private or NHS sector on prostate cancer stage and treatment | Substantial socioeconomic differences in stage and treatment patterns remain across centres in the NHS and diagnosis in private setting is associated with early stage disease and higher treatment rates | Journal of Public Health | 2012 |  |  | X |  |  |  |  |  |
| Barclay | Socio-demographic variation in stage at diagnosis of breast, bladder, colon, endometrial, lung, melanoma, prostate, rectal, renal and ovarian cancer in England and its population impact | Increasing age associated with increasing likelihood of being diagnosed with an advanced stage tumour. Increasing deprivation higher likelihood of being diagnosed with an advanced stage tumour. Eliminating socio-demographic inequalities would translate to 61% of patients with the 10 studied cancers being diagnosed at stage I/II, reducing the gap by 14% | British Journal of Cancer | 2021 |  |  | X |  |  |  |  |  |
| Bates | Exploring the impact of mental capacity on breast screening for women with intellectual disabilities | Women with intellectual disability were less likely to participate in screening | Health & Social Care in the Community | 2019 |  | X |  |  |  |  |  |  |
| Bates | A population based study of variations in operation rates for breast cancer, of comorbidity and prognosis at diagnosis: failure to operate for early breast cancer in older women | Young women and some ethnic groups present with more advanced tumours. Operation rates decrease with increasing age. Women with higher Charlson comorbidity scores were less likely to receive surgery and this may be contribute to the relationship of operation rates and age. Women from ethnic minorities were more likely to present with advanced tumours | European Journal of Surgical Oncology | 2014 | X | X |  | X |  |  |  |  |
| Batty | Modifiable risk factors for prostate cancer mortality in London: forty years of follow-up in the Whitehall study | Raised blood cholesterol increased mortality risk. Blood pressure and diabetes not associated with mortality risk. Marital status and physical stature associated with mortality risk. Smoking status and physical activity were not associated with mortality risk. No association of SES with mortality risk | Cancer Causes & Control | 2011 |  | X | X |  |  |  | X |  |
| Batura | Ethnic differences in prostate cancer presentation: a time for testing advocacy | Black men were diagnosed younger. South Asians had the highest proportion with advanced disease and the least PSA test-detected cases. | World Journal of Urology | 2023 |  |  |  | X |  |  |  |  |
| Beckett | Risk factors and survival outcome for non-elective referral in non-small cell lung cancer patients--analysis based on the National Lung Cancer Audit | Rates of non-elective presentation associated with extremes of age. Comorbidity not associated with non-elective presentation. Rates of non-elective presentation increased with increasing SES. Poorer survival confirmed in those presenting non-electively. | Lung Cancer | 2014 | X | X | X |  |  |  |  |  |
| Beecroft | Major variation in hepatocellular carcinoma treatment and outcomes in England: a retrospective cohort study | Significant variation in treatment and survival rates across the country | Frontline gastroenterology | 2023 |  |  |  |  | X |  |  |  |
| Beer | Does the HPV vaccination programme have implications for cervical screening programmes in the UK? | Women who were not vaccinated were less likely to participate in screening and those from the lowest SES areas were less likely to be vaccinated or screened | Vaccine | 2014 |  |  | X |  |  |  |  |  |
| Belot | Association between age, deprivation and specific comorbid conditions and the receipt of major surgery in patients with non-small cell lung cancer in England: A population-based study | Surgery more likely in younger people. Congestive heart failure, cerebrovascular disease and chronic obstructive pulmonary disease associated with lower surgery rates. People living in areas of lower SES less likely to have surgery. Men more likely to receive surgery than women | Thorax | 2019 | X | X | X |  |  |  |  | X |
| Benitez-Majano | Mental Health Morbidities and Time to Cancer Diagnosis Among Adults With Colon Cancer in England | People with mental health morbidity experienced a longer time to diagnosis | JAMA Network Open | 2022 |  | X |  |  |  |  |  |  |
| Berglund | Social differences in lung cancer management and survival in South East England: a cohort study | Significant reduction in operation rates with increasing age. No association of comorbidity and surgical resection rate. No variation in stage of disease in relation to SES. Likelihood of surgery was lowest in the most deprived SES group and receipt of chemotherapy for advanced disease also differed by SES. Survival significantly better in people living in higher SES areas. | BMJ Open | 2012 | X | X | X |  |  |  |  |  |
| Bharathan | Impact of deprivation on short- and long-term outcomes after colorectal cancer surgery | Increasing deprivation associated with worse short- and long-term outcomes | British Journal of Surgery | 2011 |  |  | X |  |  |  |  |  |
| Bhayat | Bone marrow transplantation in AML, and socioeconomic class: a UK population-based cohort study | Individuals from more deprived SES groups are less likely to undergo bone marrow transplantation | BMC Cancer | 2010 |  |  | X |  |  |  |  |  |
| Bhopal | Does the 'Scottish effect' apply to all ethnic groups? All-cancer, lung, colorectal, breast and prostate cancer in the Scottish Health and Ethnicity Linkage Cohort Study | Across all cancers, white people had the highest directly age standardised incidence rates and ratios (DASRR). Across ethnic minority groups DASRR varied by cancer type. It was not possible to determine if SES confounded these trends due to lack of data | BMJ Open | 2010 |  |  | X | X |  |  |  |  |
| Blakey | Socio-economic patterning in early mortality of patients aged 0-49 years diagnosed with primary bone cancer in Great Britain, 1985-2008 | Early mortality was more common in people from those living in deprived areas. | Cancer Epidemiology | 2018 |  |  | X |  |  |  |  |  |
| Blanks | Nationwide bowel cancer screening programme in England: cohort study of lifestyle factors affecting participation and outcomes in women | Acceptance of first Bowel cancer screening invitations is lower in South Asian and Black patients than white patients. Participation in screening was lower in women from socioeconomically deprived areas but FOBT positivity and adenoma detection higher | British Journal of Cancer | 2015 |  |  | X | X |  |  |  |  |
| Bone | Inequalities in the care experiences of patients with cancer: analysis of data from the National Cancer Patient Experience Survey 2011-2012 | Younger people were less likely to rate their overall care as excellent or very good. Females were less likely than males to rate their overall care as excellent of very good. People with long-standing conditions (particularly learning disabilities or mental health conditions) reported poorer care. People from Non-White ethnic groups were less likely to rate their overall case as excellent or very good | BMJ Open | 2014 | X | X |  | X |  |  |  | X |
| Borowski | Volume-outcome analysis of colorectal cancer-related outcomes | Medium and high caseload surgeons had lower anastomotic leak rates, and perioperative mortality rates than low case load surgeons. Medium and high caseload surgeons had better 5-year survival for rectal patients than those with a low caseload | British Journal of Surgery | 2010 |  |  |  |  | X |  | X |  |
| Bourkiza | Role of ethnicity and socioeconomic status (SES) in the presentation of retinoblastoma: findings from the UK | Older age at presentation associated with enucleation, bilateral disease and use of adjuvant chemotherapy. South Asian ethnicity associated with a higher likelihood of presentation with advanced disease, but effect not statistically significant. People from lower SES groups had a higher likelihood of presentation with advanced disease, but effect not statistically significant | BMJ Open Ophthalmology | 2020 |  |  | X | X |  |  |  |  |
| Boyce | Inequalities in health? An update on the effect of social deprivation for patients with breast cancer in South East Wales | Rates of early cancer detection were lower in people residing in more socioeconomically deprived areas | The Surgeon | 2019 |  |  | X |  |  |  |  |  |
| Boyd | Gender disparities in the tumor genetics and clinical outcome of multiple myeloma | There are sex-related differences in genetic events leading to the occurrence, and potentially the outcome, of myeloma | Cancer Epidemiology, Biomarkers & Prevention | 2011 |  |  |  |  |  |  |  | X |
| Boyle | Determinants of Variation in the Use of Adjuvant Chemotherapy for Stage III Colon Cancer in England | Chemotherapy use more likely in younger people. Chemotherapy use more likely in people with lower comorbidity. Chemotherapy use more likely in higher SES groups. Significant variation across providers in the use of chemotherapy | Clinical Oncology | 2020 | X | X | X |  |  |  |  |  |
| Bradley | Associations between general practice characteristics and chest X-ray rate: An observational study | Chest X-ray use was more common in general practices with more people over the age of 65. Higher practice scores for Asian and mixed ethnic groups were associated with increased chest X-ray use. Greater proportions of patients of Black ethnicity were associated with lower chest X-ray use. Chest X-ray use was less common in general practices with higher proportions of men | British Journal of General Practice | 2022 | X |  | X | X |  |  |  | X |
| Brewster | Characteristics of patients dying within 30 days of diagnosis of breast or colorectal cancer in Scotland, 2003-2007 | Those dying rapidly were more likely to be elderly. Early mortality often caused by diseases other than cancer so may be due to comorbidity | British Journal of Cancer | 2011 | X | X |  |  |  |  |  |  |
| Bright | Inequalities in colorectal cancer screening uptake in Wales: an examination of the impact of the temporary suspension of the screening programme during the COVID-19 pandemic | Post-pandemic, no decline in uptake in older individuals and uptake remains low in younger people. Uptake remains low in ethnic minority groups. Post-pandemic, no decline in uptake in the most income deprived groups but uptake remains lower in these populations. Uptake remains low in males | BMC Public Health | 2023 | X |  | X | X | X |  |  | X |
| Bright | Does geodemographic segmentation explain differences in route of cancer diagnosis above and beyond person-level sociodemographic variables? | Greater levels of deprivation associated with greater odds of emergency presentation | Journal of Public Health | 2021 |  |  | X |  |  |  |  |  |
| Brocklehurst | Analysis of the impact of deprivation on urgent suspected head and neck cancer referrals in the Mersey region between January 2004 to December 2006 | Individuals living in more socioeconomically deprived areas were more likely to experience diagnostic delays | The British Journal of Oral & Maxillofacial Surgery | 2012 |  |  | X |  |  |  |  |  |
| Brogden | The Role of Demographics, Social Deprivation and Ethnicity on Anal Squamous Cell Carcinoma Incidence in England | Black Caribbean and African patients are more likely to present with anal cancer at an earlier age with later staging. Anal cancer diagnosis at an early age was associated with lower socioeconomic groups. Female incidence of anal cancer increasing more rapidly than that of males. Males more likely than females to be diagnosed with early stage disease. | Journal of Clinical Medicine | 2021 |  |  | X | X |  |  |  |  |
| Brown | Cancer mortality 1981-2016 and contribution of specific cancers to current socioeconomic inequalities in all cancer mortality: A population-based study | Relative inequalities in total cancer mortality driven by inequalities in lung cancer. Mortality rates of lung cancer have halved for males since 1981 but doubled for females. Overall, cancer mortality has fallen by 24% for males and 10% for females. | Cancer Epidemiology | 2021 |  |  | X |  |  |  |  |  |
| Brown | Prevalence of cancer risk factors among transgender and gender diverse individuals: a cross-sectional analysis using UK primary care data | Multiple cancer risk factors are more prevalent among TGD individuals compared with cisgender individuals | British Journal of General Practice | 2023 |  |  |  |  |  | X |  |  |
| Burt | Equity of use of specialist palliative care by age: cross-sectional study of lung cancer patients | Age was not associated with access to specialist palliative care. Comorbidity was not associated with access to specialist palliative care. SES was not associated with access to specialist palliative care. Sex was not associated with access to specialist palliative care | Palliative Medicine | 2020 | X |  |  |  |  |  |  |  |
| Burton | Regional variations in hepatocellular carcinoma incidence, routes to diagnosis, treatment and survival in England | Increasing age associated with increasing incidence and reduced likelihood of treatment. Geographical variation in incidence, emergency presentation rates and survival. Curative treatment rates lower in more deprived groups | British Journal of Cancer | 2022 |  |  |  |  | X |  |  |  |
| Butler | Fatal prostate cancer incidence trends in the United States and England by race, stage, and treatment | Black men have higher fatal prostate incidence than white men | British Journal of Cancer | 2020 |  |  |  | X |  |  |  |  |
| Byrne | Inequalities in Implementation and Different Outcomes During the Growth of Laparoscopic Colorectal Cancer Surgery in England: A National Population-Based Study from 2002 to 2012 | Laparoscopic surgery use lower in people with greater comorbidity. Laparoscopic surgery use lower in people from more deprived areas | World Journal of Surgery | 2018 |  | X | X |  |  |  |  |  |
| Cameron | The impact of bivalent HPV vaccine on cervical intraepithelial neoplasia by deprivation in Scotland: reducing the gap | HPV vaccination is effective in reducing CIN and the effect is strongest in women from the most deprived areas | Journal of Epidemiology and Community Health | 2017 |  |  | X |  |  |  |  |  |
| Campbell | Are there ethnic and religious variations in uptake of bowel cancer screening? A retrospective cohort study among X.7 million people in Scotland | Men of South Asian descent have lower uptake of colorectal screening than White or Chinese men. Variation in uptake was also observed by religion, with lower rates among Hindu, Muslim and Sikh compared with the Church of Scotland. Variation in screening uptake across ethnic groups differs for males and females | BMJ Open | 2020 |  |  |  | X |  |  | X |  |
| Campbell | Exploring differences in referrals to a hospice at home service in two socio-economically distinct areas of Manchester, UK | Referral to hospice services were significantly lower in more deprived groups | Palliative Medicine | 2010 |  |  | X |  |  |  |  |  |
| Carney | Income inequality in uptake of voluntary versus organised breast cancer screening: evidence from the British Household Panel Survey | An organised breast screening programme is likely to reduce income related inequality over a screening programme where women must organise their own screen. | BMC Public Health | 2018 |  |  | X |  |  |  |  |  |
| Caulley | Association between socioeconomic indicators and geographic distribution of vestibular schwannomas in West Scotland: a 15-year review | Incidence lower in people from European Union accession countries. Incidence lowest in cases with level X qualifications. No correlation with other SES risk factors | Journal of Laryngology & Otology | 2020 |  |  | X | X |  |  |  |  |
| Chadwick | Cervical screening uptake and rates of cervical dysplasia in the British Society for Rheumatology Biologics Register for Rheumatoid Arthritis | Women with rheumatoid arthritis have higher cervical screening uptake than the general population | Rheumatology | 2020 |  | X |  |  |  |  |  |  |
| Chambers | Factors associated with advanced colorectal cancer differ between young and older adults in England: a population-based cohort study | Tumours in younger people more likely to present as advanced. In older onset patients, Black ethnicity was associated with advanced stage presentation | Colorectal Disease | 2020 | X |  | X | X |  |  |  |  |
| Chang | A cohort study on mental disorders, stage of cancer at diagnosis and subsequent survival | No impact of mental disorders on stage at presentation. People with severe mental disorders, depression, dementia and substance use had significantly worse survival | BMJ Open | 2014 |  | X |  |  |  |  |  |  |
| *Cheng* | *Elucidating the spatially varying relation between cervical cancer and socio-economic conditions in England* | *Incidence rate and proportion of low social status population varies spatially* | *International Journal of Health Geographics* | *2011* |  |  | *X* |  |  |  |  |  |
| Cheung | Factors Associated with Upper Gastrointestinal Cancer Occurrence After Endoscopy that Did Not Diagnose Cancer | Younger age associated with occurrence of cancer after a negative endoscopy. People with greater comorbidity more likely to be diagnosed with cancer after a negative endoscopy. Greater deprivation associated with occurrence of cancer after a negative endoscopy. Females more likely to be diagnosed with cancer after a negative endoscopy | Digestive Diseases and Sciences | 2016 | X | X | X |  |  |  |  | X |
| Cheyne | Social deprivation does not affect lung cancer stage at presentation or disease outcome | SES does not affect stage at diagnosis, performance status, treatment or survival | Lung Cancer | 2013 |  |  | X |  |  |  |  |  |
| Clark | Interval cancers in a national colorectal screening programme based on faecal immunochemical testing: Implications for faecal haemoglobin concentration threshold and sex inequality | Interval cancer rates were higher in women than men | Journal of Medical Screening | 2023 |  |  |  |  |  |  |  | X |
| Clucas | Cancer patients' respect experiences in relation to perceived communication behaviours from hospital staff: analysis of the 2012-2013 National Cancer Patient Experience Survey | The likelihood of perceived respect were lower for people with a long-standing physical or mental health condition. Unemployed people were more likely to report negative experiences around respect and dignity. There were differences in respect experience in relation to perceived communications by sex | Supportive Care in Cancer | 2016 |  | X | X | X |  |  |  | X |
| Conway | Accessing cancer services in North West England: the Chinese population | Chinese patients under use cancer prevention programmes and tertiary cancer services. A significant proportion of this population is dissatisfied with the service and lack confidence in it. | European Journal of Cancer Care | 2014 |  |  |  | X |  |  |  |  |
| Conway | Components of socioeconomic risk associated with head and neck cancer: a population-based case-control study in Scotland | People living in more deprived areas, and who were unemployed had a higher risk of cancer compared to those with high levels of educational attainment but effect was lost when adjustments were made of smoking and consumption of alcohol | The British Journal of Oral & Maxillofacial Surgery | 2010 |  |  | X |  |  |  |  |  |
| Copson | Ethnicity and outcome of young breast cancer patients in the United Kingdom: the POSH study | Young Black women have significantly poorer outcomes than white women. Black ethnicity is an independent risk factor for reduced distant relapse free survival particularly in oestrogen receptor positive patients | British Journal of Cancer | 2014 |  |  |  | X |  |  |  |  |
| Coupland | Ethnicity in relation to incidence of oesophageal and gastric cancer in England | Variation in incidence of cancer across ethnic groups. Black people had a higher incidence of gastric cancer than White people. White men had a higher incidence of oesophageal and gastric cardia cancers compared to other groups. White men had a higher incidence of oesophageal cancer than other groups. Bangladeshi women had a higher incidence of oesophageal cancer than white women. | British Journal of Cancer | 2012 |  |  |  | X |  |  |  |  |
| Coupland | Does place of death from cancer vary between ethnic groups in South East England? | Death in a hospice significantly less likely for Pakistani, Indian, and Bangladeshi patients. Death at home significantly less likely for Black African, Black Caribbean, and Chinese patients. Pakistani, Indian, Bangladeshi, Black African, Black Caribbean, and Chinese patients all more likely than White patients to die in hospital. | Palliative Medicine | 2011 |  |  |  | X |  |  |  |  |
| Craigs | Older age is associated with less cancer treatment: a longitudinal study of English cancer patients | People over 80 were less likely to receive chemotherapy, radiotherapy, a hospital palliative care referral or be admitted to hospital but were more likely to die in a care home | Age and Ageing | 2018 | X |  |  |  |  |  |  |  |
| Craigs | Access to hospital and community palliative care for patients with advanced cancer: A longitudinal population analysis | People over 80 years of age were less likely to access palliative care. Males were less likely to access palliative care | PLoS ONE | 2018 | X |  |  |  |  |  |  | X |
| Cranfield | Primary care blood tests before cancer diagnosis: National Cancer Diagnosis Audit data | Pre-cancer diagnosis blood testing was more common in older people. Pre-cancer diagnosis blood testing was less common in black and ethnic minority populations compared to white groups. Pre-cancer diagnosis blood testing was less common in females than males | British Journal of General Practice | 2022 | X |  |  | X |  |  |  | X |
| Crawford | Social and geographical factors affecting access to treatment of colorectal cancer: A cancer registry study | No effect of travel time on treatment. People in most deprived quartile were significantly more likely to be diagnosed at stage 4 for rectal cancer, but less so for colonic cancer. Patients in the most deprived quartile were less likely to receive chemotherapy for stage 4 disease. | BMJ Open | 2012 |  |  | X |  | X |  |  |  |
| Crawford | Cancer of Unknown Primary: a Cancer Registry Study of Factors Affecting Access to Diagnosis | Histological diagnosis declined with increasing age. Histological diagnosis rates decreased with increasing travel time to hospital. Lower socioeconomic status was associated with lower levels of histological confirmation. No association of histological diagnosis with sex | Clinical oncology | 2017 | X |  | X |  | X |  |  | X |
| Creavin | Inequality in uptake of bowel cancer screening by deprivation, ethnicity and smoking status: cross-sectional study in 86 850 citizens | Uptake was lower in ethnic minority groups. Uptake was lower in more deprived groups. Screening uptake was lower in people who smoked | Journal of Public Health | 2023 |  |  | X | X |  |  | X |  |
| Cromie | Socio-economic and ethnic disparities in childhood cancer survival, Yorkshire, UK | Differences in leukaemia survival by ethnic group were seen to be decreasing but increasing in children with central nervous system tumours and lymphoma. Increasing deprivation was associated with poor survival for children with leukaemia | British Journal of Cancer | 2023 |  |  | X | X |  |  |  |  |
| Csikar | Incidence of oral cancer among South Asians and those of other ethnic groups by sex in West Yorkshire and England, 2001-2006 | Oral cancer is more common among South Asian women than those of other ethnic groups. Men in other ethnic groups had a higher incidence than those from South Asia | The British Journal of Oral & Maxillofacial Surgery | 2013 |  |  |  | X |  |  |  |  |
| Cummings | Comorbidities are associated with poorer quality of life and functioning and worse symptoms in the 5 years following colorectal cancer surgery: Results from the ColoREctal Well-being (CREW) cohort study | Presence of any limiting comorbidities was associated with worse health related quality of life but the effect was biggest for depression and anxiety | Psycho-Oncology | 2018 |  | X |  |  |  |  |  |  |
| Curran | The association between deprivation and the incidence and survival of patients with hepatocellular carcinoma in the West of Scotland | Incidence of hepatocellular carcinoma was higher in people living in more deprived areas. There was no survival difference in relation to socioeconomic status | Expert Review of Gastroenterology & Hepatology | 2021 |  |  | X |  |  |  |  |  |
| Damery | Colorectal cancer screening using the faecal occult blood test (FOBt): a survey of GP attitudes and practices in the UK | Positive attitudes were associated with personal experience of screening and Asian ethnicity. GPs from practices in more deprived areas were more likely to have positive attitudes towards screening | BMC Family Practice | 2010 |  |  | X | X |  |  |  |  |
| Davies | Investigation of low 5-year relative survival for breast cancer in a London cancer network | The low survival rate in the Cancer Network was due to late diagnosis of patients | British Journal of Cancer | 2010 |  |  | X |  |  |  |  |  |
| Davies | Socioeconomic and ethnic inequalities in screen-detected breast cancer in London | Indian women with have higher odds of screen-detected breast cancer than white women, while Black Caribbean and African women have significantly lower odds. The incidence of screen-detected breast cancer was lower in deprived women and their 5-year relative survival was worse than affluent women. | Journal of Public Health | 2013 |  |  | X | X |  |  |  |  |
| Day | Changes in lung cancer incidence in South Asians in Leicester, 1990-2005 | Over the time periods studied the risk of cancer increased in South Asian men whereas it fell in non-South Asian men. There was also a significant rise in incidence in non-South Asian women | Journal of Public Health | 2010 |  |  |  | X |  |  |  |  |
| Deane | Who Presents Where? A Population-Based Analysis of Socio-Demographic Inequalities in Head and Neck Cancer Patients' Referral Routes | People over the age of 65 were more likely to present as an emergency. Non-white older people were more likely to present as an emergency. People living in urban areas were more likely to present as an emergency. Higher levels of deprivation were associated with more emergency presentation and increased use of the two week wait. Dental referral was more likely in women, with oral cancers and lower stage disease | International Journal of Environmental Research and Public Health | 2022 | X |  | X | X | X |  |  | X |
| Dejardin | The influence of geographical access to health care and material deprivation on colorectal cancer survival: evidence from France and England | Travel times to hospital not associated with survival. Decreasing survival associated with increasing deprivation | Health & Place | 2014 |  |  | X |  | X |  |  |  |
| Delon | Differences in cancer incidence by broad ethnic group in England, 2013-2017 | Incidence rates for most cancer sites are lower in non-white ethnic groups than white groups, though there are some exceptions in specific cancers: higher prostate cancer and Hodgkin’s lymphoma in Black men, higher myeloma, thyroid and gastrointestinal cancers in Black people. Additionally higher incidence of gastrointestinal and thyroid cancer in Asian people, and higher Hodgkin’s lymphoma in Asian men. | British Journal of Cancer | 2022 |  |  |  | X |  |  |  |  |
| Dickson | Uptake of invitations to a lung health check offering low-dose CT lung cancer screening among an ethnically and socioeconomically diverse population at risk of lung cancer in the UK (SUMMIT): a prospective, longitudinal cohort study | Uptake increased with increasing age. Uptake was higher among Chinese, Indian and other Asian ethnicities compared to those with who were White British. Uptake was lower among those of other White ethnicity compared to White British. Uptake was lower in lower SES groups. Uptake was lower in males than females | The Lancet- Public health | 2023 | X |  | X | X |  |  |  | X |
| Digby | Deprivation and faecal haemoglobin: implications for bowel cancer screening | As deprivation increased, faecal haemoglobin increased. This must be considered when selecting appropriate cut offs for positive screening tests and supports including deprivation in risk-scoring systems | Journal of Medical Screening | 2014 |  |  | X |  |  |  |  |  |
| Din | Age and Gender Variations in Cancer Diagnostic Intervals in 15 Cancers: Analysis of Data from the UK Clinical Practice Research Datalink | Mean diagnostic interval was greater for older patients in bladder, kidney, leukaemia, lung and colorectal cancer. Mean diagnostic interval was shorter with increasing age in gastric and pancreatic cancer. Diagnostic intervals were longer for females compared to males in 6 gender non-specific cancers (bladder, colorectal, gastric, head and neck, lung and lymphoma) | PLoS One | 2015 | X |  |  |  |  |  |  | X |
| Doherty | Trends in skin cancer incidence by socioeconomic position in Scotland, 1978-2004 | Skin cancer incidence is increasing but is greatest in those living in the least deprived areas | British Journal of Cancer | 2010 |  |  | X |  |  |  |  |  |
| Donaldson | Oral cavity, pharyngeal and salivary gland cancer: disparities in ethnicity-specific incidence among the London population | Compared with White patients, the highest incidence rate ratios of nasopharyngeal cancer is seen in Chinese men and women. Waldeyer's ring cancers are most common in Bangladeshi and White groups. | Oral Oncology | 2012 |  |  |  | X |  |  |  |  |
| Donkers | Socioeconomic deprivation and survival in endometrial cancer: The effect of BMI | Socioeconomic deprivation is associated with survival in endometrial cancer patients but after adjusting for confounders this association does not remain. No evidence of association of socioeconomic deprivation with peri-operative outcomes | Gynaecologic Oncology | 2020 |  |  | X |  |  |  |  |  |
| Donkers | The impact of socioeconomic deprivation on mortality in cervical cancer patients in Cornwall (England) | No association of SES with mortality, recurrence of disease or chemotherapy or radiotherapy related toxicity | European Journal of Cancer Care | 2021 |  |  | X |  |  |  |  |  |
| Donnelly | Predictors of an early death in patients diagnosed with colon cancer: a retrospective case-control study in the UK | Living in the most deprived areas, being unmarried and living alone were predictors of early death from colon cancer | BMJ Open | 2019 | X |  | X |  | X |  |  |  |
| *Donnelly* | *Socio-economic inequalities in cancer incidence - the choice of deprivation measure matters* | *While the choice of time period for measuring area-based deprivation makes little difference to relative inequalities, smaller geographic units and income based deprivation measures can produce a stronger relationship between deprivation and cancer incidence than other measures.* | *Cancer Epidemiology* | *2011* |  |  | *X* |  |  |  |  |  |
| Douglas | Socioeconomic inequalities in breast and cervical screening coverage in England: are we closing the gap? | Over time socioeconomic differences in screening participation for breast cancer have reduced but not for cervical screening | Journal of Medical Screening | 2016 |  |  | X |  |  |  |  |  |
| Douglas | Colposcopy attendance and deprivation: A retrospective analysis of 27,193 women in the NHS Cervical Screening Programme | Women from more deprived areas were less likely to attend at colposcopy | British Journal of Cancer | 2015 |  |  | X |  |  |  |  |  |
| *Downing* | *Latent class modelling of the association between socioeconomic background and breast cancer survival status at 5 years incorporating stage of disease* | *Increasing deprivation was associated with more advanced stage at diagnosis and poorer survival* | *Journal of Epidemiology and Community Health* | *2010* |  |  | *X* |  |  |  |  |  |
| Downing | *Using routinely collected health data to investigate the association between ethnicity and breast cancer incidence and survival: what is the impact of missing data and multiple ethnicities?* | *Breast cancer incidence was lower in the South Asian population compared to the White population. After adjustment for casemix, there was no difference in survival across different ethnic population groups although survival was higher in the other ethnic group depending on how ethnicity assigned. The impact of method of allocation of ethnicity had minimal impact* | *Ethnicity & Health* | *2011* |  |  |  | *X* |  |  |  |  |
| Dragoi | CAR T treatment access and outcomes in patients with large B‐cell lymphoma according to ethnicity and socioeconomic deprivation | CART-related outcomes were similar regardless of socioeconomic status or ethnicity, though deprivation is linked to inferior survival and access. | Haematological Oncology | 2023 |  |  | X | X |  |  |  |  |
| Dumas | Under-Treatment of Older Patients with Newly Diagnosed Epithelial Ovarian Cancer Remains an Issue | Increasing age associated with lower rates of receiving standard of care. Older women less likely to complete planned chemotherapy course | Cancers | 2021 | X |  |  |  |  |  |  |  |
| Duncan | Rural and Urban patients' Requirements and Experiences of Out-of-hours medical care after cancer (RUREO): a questionnaire study | Urban and rural dwellers have similar beliefs, attitudes towards and patterns of OOH service use. Place of residence does not influence decisions about whether to access OOH medical care | BMJ Open | 2023 |  |  |  |  | X |  |  |  |
| Dunlop | Evaluation of the association of area-level socioeconomic deprivation and breast cancer recurrence by oestrogen receptor subtypes in Scotland | No significant differences in prognostic tumour characteristics or risk of ipsilateral breast cancer recurrence (IBR) with deprivation except screen detection with those residing in the more deprived areas having lower screen-detection rates. Amongst those with oestrogen receptor negative tumours the most deprived had increased 5-year IBR risk | Breast Cancer Research | 2023 |  |  | X |  |  |  |  |  |
| Ekechi | Knowledge of cervical cancer and attendance at cervical cancer screening: a survey of Black women in London | Ethnicity, migration and religiosity play a role in predicting cervical screening attendance among women from Black backgrounds. African women, those born in the UK and those who regularly attend church are most likely to put off attending | BMC Public Health | 2014 |  |  |  | X |  |  |  |  |
| El Turabi | Variation in reported experience of involvement in cancer treatment decision making: evidence from the National Cancer Patient Experience Survey | Younger patients reported less positive experiences of decision making. Ethnic minority patients report substantially less positive experiences of involvement in cancer treatment decision making. | British Journal of Cancer | 2013 | X |  |  | X |  |  |  |  |
| Elledge | A library-based ecological study to investigate the contribution of ethnicity to the incidence of oral cancer within health authorities in England and Wales | Weak association of ethnicity with oral cancer | The British Journal of Oral & Maxillofacial Surgery | 2011 |  |  |  | X |  |  |  |  |
| Ellis | Inequalities in cancer survival: Spearhead Primary Care Trusts are appropriate geographic units of analyses | Spearhead primary care trusts (PCT) and spearhead local authorities (LA) are suitable geographic units via which to assess SES inequalities | Health Statistics Quarterly | 2010 |  |  |  |  | X |  |  |  |
| Ellis | How many deaths would be avoidable if socioeconomic inequalities in cancer survival in England were eliminated? A national population-based study, 1996-2006 | Survival rates improved over time but the gap between different socioeconomic groups is not changing. 11% of cancer deaths would be avoided if the SES differences could be eliminated | European Journal of Cancer | 2012 |  |  | X |  |  |  |  |  |
| Elnaggar | Experience of a District General Hospital With a Diverse Community in Operated Colorectal Cancers According to Ethnic Background | Emergency presentation was the highest in White non-UK patients. There were differences in tumour site, development of metastases and site of tumour recurrence by ethnic group. | Cureus | 2023 |  |  |  | X |  |  |  |  |
| Evans | Sporadic implementation of UK familial mammographic surveillance guidelines 15 years after original publication | Geographic inequities in the provision of breast cancer screening persist | British Journal of Cancer | 2020 |  |  |  |  | X |  |  |  |
| Evans | Breast cancer risk in a screening cohort of Asian and white British/Irish women from Manchester UK | Asian women attending screening are likely to have lower breast cancer risk than white women. | BMC Public Health | 2018 |  |  |  | X |  |  |  |  |
| Evans | Clinical presentation and initial management of black men and white men with prostate cancer in the United Kingdom: the PROCESS cohort study | No evidence of differences in disease characteristics at the time of prostate cancer diagnosis, nor of under-investigation or under-treatment of Black men compared to White men | British Journal of Cancer | 2010 |  |  |  | X |  |  |  |  |
| Evans | Differential trends in the rising incidence of endometrial cancer by type: data from a UK population-based registry from 1994 to 2006 | Increase in incidence is greatest in the 60-79 age group. Incidence is increasing across all SES groups | British Journal of Cancer | 2011 | X |  | X |  |  |  |  |  |
| Exarchakou | Colorectal cancer incidence among young adults in England: Trends by anatomical sub-site and deprivation | Incidence increases of tumours of the right colon were greatest in affluent young adults | PloS One | 2019 | X |  | X |  |  |  |  |  |
| *Exarchakou* | *Socio-economic inequalities in cancer survival: how do they translate into Number of Life-Years Lost?* | *The largest SES inequalities were seen in adults <45 years with poor prognosis cancers. For moderate/good prognosis cancers SES inequalities widen with age. More life years are lost in more deprived young people with cancer* | *British Journal of Cancer* | *2022* | *X* |  | *X* |  |  |  |  |  |
| Exarchakou | Impact of national cancer policies on cancer survival trends and socioeconomic inequalities in England, 1996-2013: population based study | Deprivation gaps in cancer survival persist and have been unaffected by national cancer policy | British Medical Journal | 2018 |  |  | X |  |  |  |  |  |
| Eylert | Falling bladder cancer incidence from 1990 to 2009 is not producing universal mortality improvements | Highest incidence and mortality is seen in industrial areas. Gender differences in incidence decreased though the deprivation gap remained unchanged. Mortality increased in older males | Journal of Clinical Urology | 2014 | X |  | X |  | X |  |  | X |
| Eylert | The impact of socio-economic deprivation on incidence, treatment and mortality from prostate cancer in England, 1990-2010 | Incidence increasing in all SES groups, but highest amongst the least deprived. Survival is consistently better in the least deprived. Prostatectomies are more frequent in the least deprived groups. | Journal of Clinical Urology | 2016 |  |  | X |  |  |  |  |  |
| Fairley | Access to principal treatment centres and survival rates for children and young people with cancer in Yorkshire, UK | South Asians had an increased risk of death for lymphoma. Increasing deprivation was significantly associated with an increased risk of death for germ cell tumours only. No significant differences in survival by sex | BMC Cancer | 2017 |  |  | X | X |  |  |  | X |
| Finlayson | Incidence of thyroid cancer in England by ethnic group, 2001-2007 | The risk of thyroid cancer varies significantly across ethnic groups and is greater for most ethnic minorities | British Journal of Cancer | 2014 |  |  |  | X |  |  |  |  |
| Floud | Disability and participation in breast and bowel cancer screening in England: a large prospective study | Participation in routine cancer screening programmes in England is reduced in people with disabilities and participation varies by type of disability | British Journal of Cancer | 2017 |  | X |  |  |  |  |  |  |
| Forbes | Breast cancer awareness and barriers to symptomatic presentation among women from different ethnic groups in East London | South Asian and black women had lower breast cancer awareness than white women. South Asian women, reported more emotional barriers to seeking medical help than white women. White women were more likely than non-white women to report worry about wasting the doctor’s time as a barrier to symptomatic presentation | British Journal of Cancer | 2011 |  |  |  | X |  |  |  |  |
| Forrest | The role of receipt and timeliness of treatment in socioeconomic inequalities in lung cancer survival: population-based, data-linkage study | Socioeconomic inequalities in survival from lung cancer were statistically explained by socioeconomic inequalities in receipt of treatment, but not by timeliness of referral and treatment. | Thorax | 2015 |  |  | X |  |  |  |  |  |
| Forrest | Factors associated with timeliness of post-primary care referral, diagnosis and treatment for lung cancer: population-based, data-linkage study | Older people waited longer for treatment. Patient who appeared ill were referred, diagnosed and treated more quickly | British Journal of Cancer | 2014 | X | X |  |  |  |  |  |  |
| Forrest | The role of patient, tumour and system factors in socioeconomic inequalities in lung cancer treatment: population-based study | The likelihood of surgery was lower in the most deprived group but linked to histological sub-type of tumour. Chemotherapy use was lower in the most deprived group but linked to poor performance status. | British Journal of Cancer | 2014 |  |  | X |  |  |  |  |  |
| Forster | Prostate cancer in the British Asian population: A case-control study | South Asian patients had less screen-detected prostate cancer, more co-morbidity, and more high-risk-disease than White patients. South Asian men more likely to under go surgery than White men | Journal of Clinical Urology | 2021 |  |  |  | X |  |  |  |  |
| Fowler | Persistent inequalities in 90-day colon cancer mortality: an English cohort study | 90-day mortality increased with increasing deprivation | British Journal of Cancer | 2017 |  |  | X |  |  |  |  |  |
| Franklyn | Geographical variations in long term colorectal cancer outcomes in England: a contemporary population analysis revealing the north-south divide in colorectal cancer survival | Significant variation in survival across England | Surgical Endoscopy | 2023 |  |  |  |  | X |  |  |  |
| Frobisher | Employment status and occupational level of adult survivors of childhood cancer in Great Britain: The British childhood cancer survivor study | Gender, age, and epilepsy were associated with employment in cancer survivors | International Journal of Cancer | 2017 |  |  | X |  |  |  |  |  |
| Fry | Relationship between ethnicity and stage at diagnosis in England: a national analysis of six cancer sites | Caribbean, African and Asian women with breast or ovarian cancer, Caribbean and African women with uterine or colon cancer, Caribbean women with NSCLC and Caribbean men with colon cancer had increased odds of late-stage disease at diagnosis compared with white British cohort. Caribbean and African men with prostate cancer had decreased odds of late stage cancer | BMJ Open | 2023 |  |  |  | X |  |  |  |  |
| Galm | Thyroid cancer: is ethnicity relevant? | Asian women presented with thyroid cancer at a significantly younger mean age than White women | The Journal of Laryngology and Otology | 2011 |  |  |  | X |  |  |  |  |
| Gathani | Ethnic differences in breast cancer incidence in England are due to differences in known risk factors for the disease: prospective study | South Asian and Black women in England have lower incidence rates of breast cancer than White women largely explained by differences in risk factors | British Journal of Cancer | 2014 |  |  |  | X |  |  |  |  |
| Gathani | Ethnicity and the surgical management of early invasive breast cancer in over 164 000 women | Allowing for different patterns of age and stage at presentation, the surgical management of early breast cancer is similar in all women, regardless of ethnicity | British Journal of Surgery | 2021 |  |  |  | X |  |  |  |  |
| Gathani | Ethnicity and the tumour characteristics of invasive breast cancer in over 116,500 women in England | All ethnic minority women with breast cancer, apart from Indian women, have greater odds than white women with breast cancer of less favourable tumour characteristics. These differences were greater in older women | British Journal of Cancer | 2021 |  |  |  | X |  |  |  |  |
| Gatrell | Variation in geographic access to specialist inpatient hospices in England and Wales | People residing in urban areas have relatively good access, large parts of England and Wales have poor access. | Health & Place | 2012 |  |  |  |  | X |  |  |  |
| Gavin | Trends in skin cancer knowledge, sun protection practices and behaviours in the Northern Ireland population | Lower use of sunscreen amongst the unemployed which may suggest cost is an issue. Lower levels of reported self-examination of skin in those with lower educational attainment. Sunscreen use more common in females than males. Skin self-examination was infrequent and less common in males than females | European Journal of Public Health | 2012 |  |  | X |  |  |  |  | X |
| Geddes | Colorectal cancer screening participation: Exploring relationship heterogeneity and scale differences using multiscale geographically weighted regression | Deprivation was found to have a strong negative impact on participation. Ethnic population concentration was associated with male participation | Geospatial Health | 2021 |  |  | X | X |  |  |  |  |
| Ghoubara | Black women with postmenopausal bleeding have lower prevalence of endometrial cancer than other ethnic groups | Lower incidence of endometrial cancer in Black women | Climacteric | 2019 |  |  |  | X |  |  |  |  |
| Gibson | The association of smoking and socioeconomic status on cutaneous melanoma: a population-based, data-linkage, case-control study | Patients with higher socioeconomic status had an increased association with melanoma incidence but better overall and disease-specific survival. | British Journal of Dermatology | 2020 |  |  | X |  |  |  |  |  |
| Gildea | Thirty-day postoperative mortality for endometrial carcinoma in England: a population-based study | Post-operative mortality rates were higher in older women. One hospital had higher mortality rates than expected after adjusting for patient case mix | British Journal of Obstetrics and Gynaecology | 2011 | X |  |  |  | X |  |  |  |
| Godbole | Impact of socioeconomic deprivation on short-term outcomes and long-term overall survival after colorectal resection for cancer | Deprivation was associated with higher postoperative complications and longer hospital stay following major resection for colorectal cancer. Its relationship with survival was not statistically significant. | International Journal of Colorectal Disease | 2019 |  |  | X |  |  |  |  |  |
| Gorman | Breast screening uptake in Polish women in Scotland | Polish women have a lower uptake of screening than the general population. | Diversity & Equality in Health and Care | 2016 |  |  |  | X |  |  |  |  |
| Green | Does patients' place of residence affect the type of physician performing primary excision of cutaneous melanoma in northern Scotland? | People living in rural areas were more likely to have excisions performed in primary care. No difference between urban and rural dwellers in Breslow thickness or completeness of excision. Those living in suburban areas and remote small towns were more likely to be treated contrary to best practice | Journal of Rural Health | 2013 |  |  |  |  | X |  |  |  |
| Grose | Variation in comorbidity and clinical management in patients newly diagnosed with lung cancer in four Scottish centers | There are inter-0center differences in investigation, treatment, and other factors | Journal of Thoracic Oncology | 2011 |  |  |  |  | X |  |  |  |
| Grose | The impact of comorbidity upon determinants of outcome in patients with lung cancer | Greater levels of comorbidity were associated with worse survival. Comorbidity levels varied between treatment centres and this impacted on survival. Patients living in more socioeconomically deprived areas had greater comorbidity levels which were associated with lower survival. Females had better survival than males | Lung Cancer | 2015 |  | X |  |  |  |  |  |  |
| Hardie | Environmental Exposures Such as Smoking and Low Vitamin D Are Predictive of Poor Outcome in Cutaneous Melanoma rather than Other Deprivation Measures | Smoking and low levels of vitamin D were predictive of survival and explained SES differences | Journal of Investigative Dermatology | 2020 |  |  | X |  |  |  |  |  |
| Haviland | Social support following diagnosis and treatment for colorectal cancer and associations with health-related quality of life: Results from the UK ColoREctal Wellbeing (CREW) cohort study | Social support was perceived to be significantly reduced in older people and this was linked to poorer health related quality of life. Social support was perceived to be significantly reduced in people living in more socioeconomically deprived areas and this was linked to poorer health related quality of life. Social support was perceived to be significantly reduced in females and this was linked to poorer health related quality of life. Social support was perceived to be significantly reduced in people with other comorbidities and this was linked to poorer health related quality of life | Psycho-Oncology | 2017 | X | X | X |  |  |  |  | X |
| Hayes | Age-related and socioeconomic inequalities in timeliness of referral and start of treatment in colorectal cancer: a population-based analysis | Older age was associated with lower likelihood of starting treatment within 31 days of diagnosis and 62 days of referral. SES was not related to referral within target but was associated with lower likelihood of starting treatment within 32 days of diagnosis or 62 days of referral | Journal of Epidemiology and Community Health | 2019 | X |  | X |  |  |  |  |  |
| Hayes | Age-related inequalities in colon cancer treatment persist over time: a population-based analysis | Older people were less likely to receive surgery and adjuvant chemotherapy. Differences narrowed over time in the younger old but did not for people >80 years | Journal of Epidemiology and Community Health | 2021 | X |  |  |  |  |  |  |  |
| Hebbar | Colorectal cancer incidence and trend in UK South Asians: a 20-year study | Lower incidence in South Asians compared with non-South Asians | Colorectal Disease | 2012 |  |  |  | X |  |  |  |  |
| Henson | Sociodemographic variation in the use of chemotherapy and radiotherapy in patients with stage IV lung, oesophageal, stomach and pancreatic cancer: evidence from population-based data in England during 2013-2014 | Comorbidity was associated with lower rates of chemotherapy but not radiotherapy. More deprived patients were consistently least likely to be treated with chemotherapy alone or chemotherapy and radiotherapy combined | British Journal of Cancer | 2018 |  | X | X |  |  |  |  |  |
| Henson | What factors influence emergency department visits by patients with cancer at the end of life? Analysis of a 124,030 patient cohort | Lower incidence in South Asians compared with non-South Asians | Palliative Medicine | 2018 | X | X | X | X |  |  |  | X |
| Hewett | Differences in intestinal metaplasia in Barrett's esophagus patients in an ethnically diverse south London population | Indian patients were less likely to have intestinal metaplasia, a precursor for oesophageal adenocarcinoma, than non-Indian patients. | Indian Journal of Gastroenterology | 2015 |  |  |  | X |  |  |  |  |
| Hirst | Uptake of the English Bowel (Colorectal) Cancer Screening Programme: an update 5 years after the full roll-out | Uptake of screening is lower in more ethnically diverse areas. Uptake was consistently lower in SES groups compared. Screening uptake is greater in females than males. | European Journal of Cancer | 2018 |  |  | X | X |  |  |  | X |
| Hounsome | Variation in usage of radical prostatectomy and radical radiotherapy for men with locally advanced prostate cancer | Radical treatment rates decreased with increasing age. Radical treatment rates decreased with increasing comorbidity. Black men half as likely as White men to receive radical treatment. Radical treatment rates decreased with increasing deprivation | Journal of Clinical Urology | 2017 | X | X | X | X |  |  |  |  |
| Hounsome | Predictors of the use of orthotopic bladder reconstruction after radical cystectomy for bladder cancer: data from a pilot study of 1756 cases 2004-2011 | Orthotopic bladder reconstruction higher in younger patients. Less frequent use of orthoptic bladder reconstruction amongst people residing in the most socioeconomically deprived areas. Orthotopic bladder reconstruction was higher in males than females | BJU International | 2013 | X |  | X |  |  |  |  | X |
| Hubbard | Cancer symptom awareness and barriers to medical help seeking in Scottish adolescents: a cross-sectional study | Anxiety was a significant independent predictor of the number of endorsed barriers to help seeking. Black and minority ethnic adolescents recognise fewer cancer warning signs. Female gender was a significant predictor of barriers to help seeking | BMC Public Health | 2014 |  | X |  | X |  |  |  | X |
| Hudson | Ethnic and age differences in right-left breast asymmetry in a large population-based screening population | Breast volume absolute asymmetry increased, whilst radiodense absolute asymmetry decreased, with increasing age. Age differences attenuated after adjustment for the corresponding underlying volumetric dimension. Relative to Whites, Blacks had statistically significantly higher, and Chinese lower, breast volume and radiodense volume absolute asymmetries. Ethnic differences attenuated after adjustment for the corresponding underlying volumetric dimension. | British Journal of Radiology | 2020 | X |  |  | X |  |  |  |  |
| Hulbert Williams | The cancer care experiences of gay, lesbian and bisexual patients: A secondary analysis of data from the UK Cancer Patient Experience Survey | Less positive care experiences reported by lesbian, gay and bisexual respondents | European Journal of Cancer Care | 2017 |  |  |  |  |  | X |  |  |
| Ingarfield | Inequality in the Survival of Patients With Head and Neck Cancer in Scotland | Survival inequalities in relation to deprivation category and highest education level explained by age, sex, health and behavioural factors. No explanatory factors fully explained survival inequalities in relation to household income | Frontiers in Oncology | 2018 |  |  | X |  |  |  |  |  |
| Ingarfield | Inequality in survival of people with head and neck cancer: Head and Neck 5000 cohort study | SES differences in cancer survival were attenuated after adjusting for other factors such as smoking and drinking | Head and Neck | 2021 |  |  | X |  |  |  |  |  |
| *Ingleby* | *Assessment of the concordance between individual-level and area-level measures of socio-economic deprivation in a cancer patient cohort in England and Wales* | *Ecological measures don't fully capture the relationship between deprivation and health* | *BMJ Open* | *2020* |  |  | *X* |  |  |  |  |  |
| Ingleby | An investigation of cancer survival inequalities associated with individual-level socio-economic status, area-level deprivation, and contextual effects, in a cancer patient cohort in England and Wales | Cancer survival associated with socioeconomic deprivation, education level and employment type. Both individual and contextual effects contribute to the differences | BMC Public Health | 2022 |  |  | X |  |  |  |  |  |
| Isherwood | Treatment Access and Survival Amongst British Asians with Pancreatic Cancer | Age at presentation is lower in Asian patients than White patients and their survival is significantly better when receiving palliative chemotherapy. | World Journal of Surgery | 2017 |  |  |  | X |  |  |  |  |
| Jack | Prostate cancer incidence, stage at diagnosis, treatment and survival in ethnic groups in South-East England | Black men had the highest incidence of prostate cancer, followed by White, then Indian/Pakistani men. The relative excess of prostate cancer in Black vs White men was strongly age-dependent. Despite differences in recorded treatment, Indian/Pakistani men had better overall survival and prostate cancer specific survival (PCSS). Black men also had better overall survival, and their PCSS was similar to that of White men | BJU International | 2010 |  |  |  | X |  |  |  |  |
| Jack | Lung cancer incidence and survival in different ethnic groups in South East England | Bangladeshi men, White men and White women had the highest incidence rates. Bangladeshi men had consistently higher survival estimates compared with White men. Indian, Black Caribbean and Black African men also had higher survival estimates. South Asian and Black women had higher survival than White women | British Journal of Cancer | 2011 |  |  |  | X |  |  |  |  |
| Jack | Breast cancer and age in Black and White women in South East England | Age specific incident rates of breast cancer are higher in white women over 50 than black women over 50. | International Journal of Cancer | 2012 | X |  |  | X |  |  |  |  |
| Jack | Differences in breast cancer hormone receptor status in ethnic groups: a London population | Black and south Asian women were more likely than White women to have the generally more aggressive triple negative breast cancer. Black women additionally had worse age-adjusted survival than White women. | European Journal of Cancer | 2013 | X |  |  | X |  |  |  |  |
| Jack | Primary liver cancer incidence and survival in ethnic groups in England, 2001-2007 | Men from any ethnic minority groups have higher liver cancer incidence than White men. Survival rates additionally vary by ethnicity. | Cancer Epidemiology | 2013 |  |  |  | X |  |  |  |  |
| Jack | Breast cancer screening uptake among women from different ethnic groups in London: a population-based cohort study | Ethnic minority women are less likely than White women to attend their first, or routine recall, screening appointment. | BMJ Open | 2014 |  |  |  | X |  |  |  |  |
| Jack | The varying influence of socioeconomic deprivation on breast cancer screening uptake in London | Screening uptake decreased with increasing deprivation | Journal of Public Health | 2016 |  |  | X |  |  |  |  |  |
| Jeevan | National trends and regional variation in immediate breast reconstruction rates | Substantial regional variation exists in immediate reconstruction rates | British Journal of surgery | 2016 |  |  |  |  | X |  |  |  |
| Jeevan | Reconstructive utilisation and outcomes following mastectomy surgery in women with breast cancer treated in England | The likelihood of being offered immediate reconstruction fell with increasing age. Women from more deprived areas experienced higher complication rates. | Annals of the Royal College of Surgeons of England | 2020 | X |  | X |  |  |  |  |  |
| Jeevan | Association between age and access to immediate breast reconstruction in women undergoing mastectomy for breast cancer | Increasing age is associated with a lower probability of an offer of immediate breast reconstruction. | British Journal of Surgery | 2017 | X |  |  |  |  |  |  |  |
| Jeevan | Socioeconomic deprivation and inpatient complication rates following mastectomy and breast reconstruction surgery | Rates of postoperative complications after mastectomy and breast reconstruction surgery were higher among women from more deprived backgrounds. | British Journal of Surgery | 2015 |  |  | X |  |  |  |  |  |
| Jeevan | Regional variation in use of immediate breast reconstruction after mastectomy for breast cancer in England | Immediate breast reconstruction is lower in older women and those with more comorbidities, as well as non-white women and those from more deprived areas. | European Journal of Surgical Oncology | 2010 | X | X | X | X |  |  |  |  |
| Jobling | Piloting a novel cancer care pathway: socioeconomic background as a barrier to access | The MDC pathway referral rate is affected by SES | Clinical Medicine | 2022 |  |  | X |  |  |  |  |  |
| Johnson | Effect of HPV vaccination and cervical cancer screening in England by ethnicity: a modelling study | The introduction of HPV vaccination in England will initially widen a pre-existing disparity in the incidence of HPV-related cancer by ethnicity, partly due to herd immunity disproportionately benefiting subgroups with high vaccination rates | The Lancet Public health | 2018 |  |  |  | X |  |  |  |  |
| Johnson | The complex relationship between household income of family caregivers, access to palliative care services and place of death: A national household population survey | Respondents’ income was unrelated to care recipients’ place of death when adjusted for palliative care access. When only caregivers were considered, decedents with caregivers from higher income quartiles were the least likely to die at home. Family caregivers from higher income brackets are likely to be powerful patient advocates | Palliative Medicine | 2018 |  |  | X |  |  |  |  |  |
| Jones | Geographical access to healthcare in Northern England and post-mortem diagnosis of cancer | No association with access to primary care but some evidence access to tertiary care may influence post-mortem diagnosis | Journal of Public Health | 2010 |  |  |  |  | X |  |  |  |
| Joyce | Factors associated with variation in emergency diagnoses of cancer at general practice level in England | Practices in more deprived areas are significantly associated with a higher proportion of emergency diagnoses of cancer. | Journal of Public Health | 2021 |  |  | X |  |  |  |  |  |
| *Kajiwara Saito* | *Socioeconomic gaps over time in colorectal cancer survival in England: flexible parametric survival analysis* | *Increasing deprivation associated with lower survival and increasing emergency diagnosis* | *Journal of Epidemiology and Community Health* | *2021* |  |  | *X* |  |  |  |  |  |
| Kanani | The association of mood disorders with breast cancer survival: an investigation of linked cancer registration and hospital admission data for South East England | Depression and bipolar disorder were, respectively, significantly and non-significantly associated with worse overall survival in breast cancer patients | Psycho-Oncology | 2016 |  | X |  |  |  |  |  |  |
| Keane | Sociodemographic trends in the incidence of pancreatic and biliary tract cancer in UK primary care | Incidence increased with age. Incidence of biliary tract cancer was significantly associated with increasing socio-economic deprivation. Incidence was higher in men | PloS One | 2014 | X |  | X |  |  |  |  | X |
| Keegan | Case-control study of paternal occupation and childhood leukaemia in Great Britain, 1962-2006 | Positive association between childhood leukaemia risk and paternal occupation involving social contact and higher paternal occupational social class. | British Journal of Cancer | 2012 |  |  | X |  |  |  |  |  |
| Keegan | Case-control study of paternal occupation and social class with risk of childhood central nervous system tumours in Great Britain, 1962-2006 | Little evidence that paternal occupation is a significant risk factor for childhood CNS tumours, either overall or for specific subtypes. Weak evidence that occupational social class of the father may be associated with childhood CNS risk | British Journal of Cancer | 2013 |  |  | X |  |  |  |  |  |
| Keeney | An exploration of public knowledge of warning signs for cancer | Older people had greater awareness of cancer symptoms. People with higher educational attainment and of higher SES had greater awareness of cancer symptoms. Females had a greater awareness of cancer symptoms | European Journal of Oncology Nursing | 2011 | X |  | X |  |  |  |  | X |
| Kelly-Irving | Childhood adversity as a risk for cancer: findings from the 1958 British birth cohort study | Odds of having cancer before 50 years of age in women was linked to experiencing adverse childhood events | BMC Public Health | 2013 |  |  |  |  |  |  | X |  |
| Kerrison | Inequalities in cancer screening participation between adults with and without severe mental illness: results from a cross-sectional analysis of primary care data on English Screening Programmes | Screening participation was lower among adults with SMI, than without. Participation was lowest in those with schizophrenia, other psychoses then bipolar disorder. Participation was lowest among people with SMI from Black ethnic groups. Participation was lowest among people with SMI who live in the most deprived areas | British Journal of Cancer | 2023 |  | X | X | X |  |  |  |  |
| Kerrison | Ethnic inequalities in older adults bowel cancer awareness: findings from a community survey conducted in an ethnically diverse region in England | Symptom awareness significantly lower in ethnic minority groups | BMC Public Health | 2021 |  |  |  | X |  |  |  |  |
| Khakwani | The impact of the 'hub and spoke' model of care for lung cancer and equitable access to surgery | Patients were more likely to be operated on if they were first seen at a surgical centre. | Thorax | 2015 |  |  |  |  | X |  |  |  |
| Kinnear | The low uptake of breast screening in cities is a major public health issue and may be due to organisational factors: a Census-based record linkage study | Screening uptake is lower in major cities. | Breast | 2011 |  |  |  |  | X |  |  |  |
| Kinnear | Are caregiving responsibilities associated with non-attendance at breast screening? | Deprived women were significantly less likely to attend screening but this was not explained by caregiving responsibilities | BMC Public Health | 2010 |  |  | X |  |  |  |  |  |
| Koffman | Does ethnicity affect where people with cancer die? A population-based 10 year study | Minority ethnic groups are more likely to die in hospital than white patients, and less likely to die in hospice or at home. | PloS One | 2014 |  |  |  | X |  |  |  |  |
| Konfortion | Time and deprivation trends in incidence of primary liver cancer subtypes in England | Liver cell carcinoma and intrahepatic bile duct carcinoma incidence increased driven by patients living in the most deprived areas. | Journal of Evaluation in Clinical Practice | 2014 |  |  | X |  |  |  |  |  |
| Korszun | Psychosocial factors associated with impact of cancer in longterm haematological cancer survivors | White ethnic groups have lower positive impact of cancer scores. Higher negative impact of cancer scores were observed in those in the more socioeconomically deprived groups. Higher negative IOC scores were significantly associated with medical comorbidity, psychological distress, lower social support, high fatigue levels and functional impairment. | British Journal of Haematology | 2014 |  | X | X | X |  |  |  |  |
| Kroll | Childhood leukaemia and socioeconomic status in England and Wales 1976-2005: evidence of higher incidence in relatively affluent communities persists over time | Childhood leukaemia incidence increases with increasing SES | British Journal of Cancer | 2011 |  |  | X |  |  |  |  |  |
| Kroll | Evidence for under-diagnosis of childhood acute lymphoblastic leukaemia in poorer communities within Great Britain | Under-diagnosis of childhood acute lymphoblastic leukaemia in areas of lower SES | British Journal of Cancer | 2012 |  |  | X |  |  |  |  |  |
| Kum | Presentation, follow-up, and outcomes among African/Afro-Caribbean men on active surveillance for prostate cancer: experiences of a high-volume UK centre | African and afro-Caribbean men have a substantially higher rate of non-attendance at scheduled follow-up visits than men of other ethnicities. | Prostate Cancer and Prostatic Diseases | 2021 |  |  |  | X |  |  |  |  |
| Lal | The impact of socioeconomic deprivation on the uptake of colorectal cancer screening in London | Screening uptake is lower in lower SES groups | Journal of Medical Screening | 2021 |  |  | X |  |  |  |  |  |
| Langton | The impact of the UK 'two-week rule' on stage-on-diagnosis of oral cancer and the relationship to socio-economic inequalities | Although stage at presentation improved over time, especially in the most deprived group, SES inequalities persisted. The introduction of the two week wait had no impact on stage at diagnosis | Journal of Cancer Policy | 2019 |  |  | X |  |  |  |  |  |
| Launders | Cancer rates and mortality in people with severe mental illness: Further evidence of lack of parity | Rate of all-cancer diagnoses was reduced in those with SMI compared to those without SMI, and particularly in those with schizophrenia. All-cause mortality after cancer was increased in the SMI group, and cancer-specific mortality was increased in those with schizophrenia | Schizophrenia Research | 2022 |  | X |  |  |  |  |  |  |
| Lejeune | Socio-economic disparities in access to treatment and their impact on colorectal cancer survival | Compared to more affluent patients, deprived patients had poorer survival were less likely to receive any treatment within six months and if treated, were more likely to receive late treatment | International Journal of Epidemiology | 2010 |  |  | X |  |  |  |  |  |
| Leonard | Impact of multiple deprivations on detection, progression and interventions in small renal masses (less than 4 cm) in a population based study | No difference across SES groups in the detection, progression or management of small renal masses <4cm | European Journal of Surgical Oncology | 2013 |  |  | X |  |  |  |  |  |
| Liao | Disparities in care and outcomes for primary liver cancer in England during 2008-2018: a cohort study of 8.52 million primary care population using the QResearch database | People aged ≥80 years were more likely to be diagnosed through emergency presentation and in late stages, less likely to receive treatments and had poorer survival than those aged <60 years. Compared with white British, Asians and Black Africans were more likely to be diagnosed with hepatocellular carcinoma. Patients with higher socioeconomic deprivation were more likely to be diagnosed through the emergency route. Men had a higher risk of being diagnosed with liver cancer than women, | EClinicalMedicine | 2023 | X |  | X | X |  |  |  | X |
| Lightfoot | Survival from childhood acute lymphoblastic leukaemia: the impact of social inequality in the United Kingdom | Significant SES disparities in acute lymphoblastic leukaemia which are not due to treatment accessibility | European Journal of Cancer | 2012 |  |  | X |  |  |  |  |  |
| Lim | The Effect of Socio-Economic Status on Severity of Periocular Basal Cell Carcinoma at Presentation | Socio-economic deprivation is associated with larger and more frequent presentation of periocular basal cell carcinoma. | Ophthalmic Plastic and Reconstructive Surgery | 2015 |  |  | X |  |  |  |  |  |
| Ling | Inequalities in treatment among patients with colon and rectal cancer: a multistate survival model using data from England national cancer registry 2012-2016 | Compared to the least deprived quintile, the most deprived with stage I–IV colorectal cancer had a lower probability of being alive and treated at all the time during follow-up, and a higher probability of being untreated and of dying | British Journal of Cancer | 2023 |  |  | X |  |  |  |  |  |
| Ling | Inequalities in cancer mortality trends in people with type 2 diabetes: 20 year population-based study in England | Between 1998-2018 cancer mortality rates decreased for 55-65 year olds but increased for 75-85 year olds. There were constant upward trends for pancreatic, liver and lung cancer at all ages, colorectal at most ages, breast cancer at younger ages and prostate and endometrial at older ages. Higher rates of increasing cancer mortality in people with morbid obesity compared to a normal body weight. People with type II diabetes had a X.5-fold increased risk of colorectal, pancreatic, liver and endometrial cancer. Increasing mortality in White people and decreasing in other ethnic groups. Higher rates of increase in mortality In the least deprived compared to the most deprived groups. Higher rates of increase in mortality in women than men | Diabetologia | 2023 | X | X | X | X |  |  |  |  |
| *Littlejohns* | *Lifestyle factors and prostate-specific antigen (PSA) testing in UK Biobank: Implications for epidemiological research* | *High BMI, being diagnosed with diabetes, heart disease or stroke were associated with less PSA testing. Vasectomy associated with higher PSA testing. Black ethnic origin is associated with a higher likelihood of prostate-specific antigen testing, whilst Asian ethnic origin is associated with lower likelihood. Increasing socioeconomic deprivation is associated with a lower likelihood of PSA testing* | *Cancer Epidemiology* | *2016* |  | *X* | *X* | *X* |  |  |  |  |
| Lloyd | Breast cancer worry in higher-risk women offered preventive therapy: a UK multicentre prospective study | Ethnic minority women were more likely to report high worry than white women. Women educated below degree level reported high worry compared to those with higher education | Breast Cancer Research and Treatment | 2021 |  |  | X | X |  |  |  |  |
| Lloyd | Lifetime risk of being diagnosed with, or dying from, prostate cancer by major ethnic group in England 2008-2010 | Black men are at twice the risk of being diagnosed with, and dying from, prostate cancer compared to White men | BMC Medicine | 2015 |  |  |  | X |  |  |  |  |
| Lloyd-Williams | Socio-economic deprivation and symptom burden in UK hospice patients with advanced cancer-findings from a longitudinal study | Patients living in the most deprived areas were significantly likely to report receiving insufficient information regarding their cancer at diagnosis, greater pain, moderate to severe depression and higher global symptom burden. | Cancers | 2021 |  |  | X |  |  |  |  |  |
| Lo | Comparing barriers to colorectal cancer screening with barriers to breast and cervical screening: a population-based survey of screening-age women in Great Britain | Age not associated with participation in screening. Single women less likely to participate in colorectal or breast screening than married women. More socioeconomically deprived women were less likely to participate in screening | Journal of Medical Screening | 2013 | X |  | X |  |  |  |  |  |
| Lo | Colorectal cancer screening uptake over three biennial invitation rounds in the English bowel cancer screening programme. | Socioeconomic deprivation is associated with gFOBT uptake | Gut | 2015 |  |  | X |  |  |  |  |  |
| Lord | Are depressive symptoms more common among British South Asian patients compared with British White patients with cancer? A cross-sectional survey | British South Asian people self-reported significantly higher rates of depressive symptoms compared with British White patients. British South Asian people reported more symptoms than other ethnic groups | BMJ open | 2013 |  |  |  | X |  |  |  |  |
| Lord | The beliefs and knowledge of patients newly diagnosed with cancer in a UK ethnically diverse population | Significantly more South Asian newly diagnosed cancer patients believe that cancer is incurable than their White patients | Clinical Oncology | 2012 |  |  |  | X |  |  |  |  |
| Lovell | Barriers to cervical screening participation in high-risk women | Women from ethnic minorities were less likely to participate | Journal of Public Health | 2015 |  |  |  | X |  |  |  |  |
| Lyman | A dermatological questionnaire for general practitioners in England with a focus on melanoma; misdiagnosis in black patients compared to white patients | GPs are less likely to correctly diagnose melanoma cases in black than white patients | Journal of the European Academy of Dermatology and Venereology | 2017 |  |  |  | X |  |  |  |  |
| Lyratzopoulos | Variation in advanced stage at diagnosis of lung and female breast cancer in an English region 2006-2009 | Younger age was associated with three or more pre-referral consultations. Three or more pre-referral more consultations was more common in ethnic minority groups. Women were more likely to have three or more pre-referral consultations than men | British Journal of Cancer | 2012 | X |  | X |  |  |  |  | X |
| Lyratzopoulos | Gender inequalities in the promptness of diagnosis of bladder and renal cancer after symptomatic presentation: evidence from secondary analysis of an English primary care audit survey | Women had three or more pre-consultation referrals more often than men and a greater delay to diagnosis | BMJ Open | 2013 |  |  |  |  |  |  |  | X |
| Lyratzopoulos | Population based time trends and socioeconomic variation in use of radiotherapy and radical surgery for prostate cancer in a UK region: continuous survey | Men from lower socioeconomic groups substantially less likely to be treated with radical surgery or radiotherapy. | British Medical Journal | 2010 |  |  | X |  |  |  |  |  |
| Lyratzopoulos | Changes over time in socioeconomic inequalities in breast and rectal cancer survival in England and Wales during a 32-year period (1973-2004): the potential role of health care | Inequalities in breast cancer survival narrowed during the study period but those in rectal cancer widened. | Annals of Oncology | 2011 |  |  | X |  |  |  |  |  |
| Lyratzopoulos | Trends in the surgical management of epithelial ovarian cancer in East Anglia 1995-2006 | Use of surgery was less frequent in most deprived compared with most affluent patients. More deprived patients were less likely to be treated with omentectomy | European Journal of Surgical Oncology | 2011 |  |  | X |  |  |  |  |  |
| MacVicar | Analysing the impact of living in a rural setting on the presentation and outcome of colorectal cancer. A prospective single centre observational study | Rural patients were more likely to be detected through screening but those who presented symptomatically were more likely to be node positive. There was no impact on survival | The Surgeon | 2020 |  |  |  |  | X |  |  |  |
| *Madden* | *Using maps and funnel plots to explore variation in place of death from cancer within London, 2002-2007* | *There was variation in place of death across primary care trusts in London* | *Palliative Medicine* | *2011* |  |  |  |  | *X* |  |  |  |
| Maheswaran | Incidence, socioeconomic deprivation, volume-outcome and survival in adult patients with acute lymphoblastic leukaemia in England | No impact of rurality on incidence or survival. Hospitals with treating low volumes had worse survival. No evidence of association between socioeconomic deprivation and incidence. Survival was decreased in more socioeconomically deprived groups. Incidence higher in males but no evidence of impact of sex on survival | BMC Cancer | 2018 | X |  | X |  | X |  |  | X |
| Maile | Nervous System and Intracranial Tumour Incidence by Ethnicity in England, 2001-2007: A Descriptive Epidemiological Study | Relative to White people South Asians, Blacks and Chinese have a lower incidence of gliomas. Blacks have a higher incidence of meningioma and pituitary tumours. There is heterogeneity in pituitary and meningioma incidence between individual South Asian ethnicities | PloS One | 2016 |  |  |  | X |  |  |  |  |
| Malcomson | Socioeconomic differences in adherence to the World Cancer Research Fund Cancer Prevention Recommendations in the UK Biobank cohort | Total adherence was not associated with socioeconomic status, but adherence to recommendations in specific areas varied by deprivation. | The Lancet | 2022 |  |  | X |  |  |  |  |  |
| Mangtani | Cancer mortality in ethnic South Asian migrants in England and Wales (1993-2003): patterns in the overall population and in first and subsequent generations | All-cancer mortality rates in South Asians were half of those in non-South Asians in first-generation and subsequent-generation South Asians The higher mortality in first-generation South Asians for liver (both sexes), oral cavity and gallbladder cancer (females), particularly marked among Bangladeshis, was reduced in subsequent generations. | British Journal of Cancer | 2010 |  |  |  | X |  |  |  |  |
| Mansouri | The impact of age, sex and socioeconomic deprivation on outcomes in a colorectal cancer screening programme | Screening uptake was lower in younger people. Individuals living in deprived areas were less likely to participate in screening, undergo colonoscopy and have a cancer identified as a result of a positive test. There was a lower uptake of screening in men than women | PloS One | 2013 | X |  | X |  |  |  |  | X |
| Marcu | Educational differences in likelihood of attributing breast symptoms to cancer: a vignette-based study | Low and mid education were associated with being less likely to attribute a nipple rash to cancer. For axillary lump, low education was associated with lower likelihood of mentioning cancer as a possible cause. Cancer avoidance was associated with lower education. | Psycho-Oncology | 2016 |  |  | X |  |  |  |  |  |
| Maringe | Cancer incidence in South Asian migrants to England, 1986-2004: unraveling ethnic from socioeconomic differentials | Age-adjusted cancer incidence in South Asians was half that of non-South Asians but rose over time. | International Journal of Cancer | 2013 |  |  |  | X |  |  |  |  |
| Maringe | Persistent inequalities in unplanned hospitalisation among colon cancer patients across critical phases of their care pathway, England, 2011-13 | Individuals from deprived areas had higher proportions of unplanned hospital admissions | British Journal of Cancer | 2018 |  |  | X |  |  |  |  |  |
| Marla | Factors influencing postoperative length of hospital stay after breast cancer surgery | No association of deprivation with post-operative length of stay | Breast | 2013 |  | X | X |  |  |  |  |  |
| Marlow | Understanding cervical screening non-attendance among ethnic minority women in England | Women from ethnic minorities were more likely to be non-attenders than White women. | British Journal of Cancer | 2015 |  |  |  | X |  |  |  |  |
| Martin | Examining the uptake of predictive BRCA testing in the UK; findings and implications | Time to testing was found to vary with age | European Journal of Human Genetics | 2021 | X |  |  |  |  |  |  |  |
| Martin | Trends in BRCA testing and socioeconomic deprivation | BRCA testing was lower in more socioeconomically deprived groups | European Journal of Human Genetics : EJHG | 2019 |  |  | X |  |  |  |  |  |
| Martins | Ethnic inequalities in routes to diagnosis of cancer: a population-based UK cohort study | Patients from Other ethnic groups were more likely to present as an emergency. Asian and Black groups were more likely to be referred by the GP and the Black and Mixed groups via the TWW | British Journal of Cancer | 2022 |  |  |  | X |  |  |  |  |
| Martins | Assessing Ethnic Inequalities in Diagnostic Interval of Common Cancers: A Population-Based UK Cohort Study | Inconsistent differences in diagnostic interval across ethnic groups | Cancers | 2022 |  |  |  | X |  |  |  |  |
| Martins | Ethnic differences in patients' preferences for prostate cancer investigation: a vignette-based survey in primary care | Black men are less likely than white men to opt for prostate cancer investigation at any risk level. | The British Journal of General Practice | 2015 |  |  |  | X |  |  |  |  |
| Martins | Are There Ethnic Differences in Recorded Features among Patients Subsequently Diagnosed with Cancer? An English Longitudinal Data-Linked Study | Compared with White patients, Asian and Black patients with breast, colorectal, and prostate cancer were more likely than White patients to have multiple features; the opposite was seen for the Black and Other ethnic groups with lung or prostate cancer. Asian and Black patients were more likely to have low-risk features (e.g., cough, upper abdominal pain) recorded. Non-White patients were less likely to have alarm features. | Cancers | 2023 |  |  |  | X |  |  |  |  |
| Maruthappu | Incidence of prostate and urological cancers in England by ethnic group, 2001-2007: a descriptive study | Non-White groups had a lower incidence of urological cancers than Whites with the exception of prostate cancer which was higher in Black men. There was strong evidence of differences in risk between Indians, Pakistanis and Bangladeshis for kidney, bladder and prostate cancer and between Black Africans and Black Caribbeans for all four cancers | BMC Cancer | 2015 |  |  |  | X |  |  |  |  |
| Mason | Neighbourhood environment and socioeconomic inequalities in cancer admissions: a prospective study using UK Biobank and linked hospital records | In deprived neighbourhoods, increasing the amount of greenspace may help reduce cancer-related hospitalizations. | Cancer Causes & Control | 2022 |  |  |  |  | X |  |  |  |
| Massat | Variation in cervical and breast cancer screening coverage in England: a cross-sectional analysis to characterise districts with atypical behaviour | Other ethnicities had the lowest coverage. Urbanisation associated with lower coverage. Increasing deprivation associated with lower coverage | BMJ Open | 2015 |  |  | X | X | X |  |  |  |
| Mastaglio | Impact of socioeconomic status on disease phenotype, genomic landscape and outcomes in myelodysplastic syndromes | If access to healthcare is equitable, socioeconomic status does not determine disease biology or survival in people with myelodysplastic syndromes receiving supportive treatment | British Journal of Haematology | 2016 |  |  | X |  |  |  |  |  |
| Maye | Do the indices of deprivation or smoking affect post-operative X-year mortality in patients undergoing a craniotomy for a brain tumour in a public healthcare system? | Increasing age associated with increasing mortality. Postcode rank not associated with mortality but those in decile X had the lowest risk of death. Males had higher mortality | Acta Neurochirurgica | 2023 | X |  | X |  |  |  |  | X |
| Mayer | The volume-mortality relation for radical cystectomy in England: retrospective analysis of hospital episode statistics | Patients treated in medium volume ones had a significantly higher odds of in-hospital and total mortality. There was a weak association suggesting higher volume surgeons had lower mortality | British Medical Journal | 2010 |  |  |  |  | X |  | X |  |
| McCowan | Comparing uptake across breast, cervical and bowel screening at an individual level: a retrospective cohort study | Increasing age was associated with increasing uptake. Decreasing social deprivation was associated with increasing uptake. Lower levels of comorbidity and previous malignancy associated with increasing uptake | British Journal of Cancer | 2019 | X | X | X |  |  |  |  |  |
| McGregor | Uptake of Bowel Scope (Flexible Sigmoidoscopy) Screening in the English National Programme: the first 14 months | Uptake of bowel screening is lower in women and in people from more socioeconomically deprived and more ethnically diverse areas. | Journal of Medical Screening | 2016 |  |  | X | X |  |  |  | X |
| McGuinness | Adherence to adjuvant endocrine therapy among White British and ethnic minority breast cancer survivors in the United Kingdom | Rates of non-adherence to adjuvant endocrine therapy in breast cancer survivors is higher in minority ethnicity women than white women. | European Journal of Cancer Care | 2022 |  |  |  | X |  |  |  |  |
| McKenzie | Socio-economic inequalities in survival from screen-detected breast cancer in South West England: population-based cohort study | The deprivation gap in survival does not appear as marked with screen-detected breast cancer as the other groups, though still apparent | European Journal of Public Health | 2012 |  |  | X |  |  |  |  |  |
| McNally | Socioeconomic patterning in the incidence and survival of children and young people diagnosed with malignant melanoma in northern England | Increased risk of melanoma was linked with some aspects of greater affluence but worse survival was associated with living in a more deprived area. | Journal of Investigative Dermatology | 2014 |  |  | X |  |  |  |  |  |
| McNally | Socioeconomic patterning in the incidence and survival of teenage and young adult men aged between 15 and 24 years diagnosed with non-seminoma testicular cancer in northern england | Increased risk of non-seminoma testicular cancer in teenage and young adult men associated lower deprivation but greater deprivation linked with worse survival | Urologic Oncology | 2015 |  |  | X |  |  |  |  |  |
| McPhail | Stage at diagnosis and early mortality from cancer in England | Excess mortality highest in older age groups. Excess mortality varied by area of residence. Greater deprivation associated with increasing excess mortality. Excess mortality higher in females in colorectal cancer and males in lung cancer | British Journal of Cancer | 2015 | X |  | X |  | X |  |  | X |
| McPhail | Emergency presentation of cancer and short-term mortality | Extremes of age associated with increased rates of emergency presentation. Greater comorbidity associated with emergency presentation. Greater deprivation associated with greater likelihood of emergency presentation | British Journal of Cancer | 2013 | X | X | X |  |  |  |  | X |
| McRonald | The UK Lung Screen (UKLS): demographic profile of first 88,897 approaches provides recommendations for population screening | Younger people were less likely to participate. Higher socioeconomic status correlated positively with response to lung cancer screening but inversely with lung cancer risk | Cancer Prevention Research | 2014 | X |  | X |  |  |  |  |  |
| McVey | Initial management of low-risk localized prostate cancer in the UK: analysis of the British Association of Urological Surgeons Cancer Registry | Treatment choice was associated with socioeconomic status. For example, radical prostatectomy was chosen more by men living in affluent areas compared to more deprived | BJU International | 2010 |  |  | X |  |  |  |  |  |
| Mehta | Socio-economic deprivation and outcomes following radical nephroureterectomy for clinically localized upper tract transitional cell carcinoma | High grade and stage disease was seen in patients from less deprived categories. No differences by SES in cancer-specific mortality and follow-up recurrences. Long-term follow-up did not show any differences in cancer-specific survival between different deprivation categories. | World Journal of Urology | 2015 |  |  | X |  |  |  |  |  |
| Mesa-Eguiagaray | Breast cancer incidence and survival in Scotland by socio-economic deprivation and tumour subtype | Deprivation is associated with differential incidence trends for screen-detected oestrogen receptor positive tumours and with higher mortality for select tumour subtypes | Breast Cancer Research and Treatment | 2022 |  |  | X |  |  |  |  |  |
| Miles | Cancer fatalism and poor self-rated health mediate the association between socioeconomic status and uptake of colorectal cancer screening in England | No association with uptake and age. Screening uptake was higher among people with better self-rated health and lower cancer fatalism. Lower depression only had an indirect effect on uptake through better self-rated health. No association with uptake and ethnicity. Uptake higher amongst people living in areas with lower deprivation. No association with uptake and gender | Cancer Epidemiology, Biomarkers & Prevention | 2011 | X | X | X | X |  |  |  |  |
| Mirza | Impact of social deprivation on the outcome of major head and neck cancer surgery in England: A national analysis | People from more socioeconomically deprived areas were younger, had higher burdens of morbidity, more frequently required emergency surgery had loner inpatient stays and higher mortality than those from more affluent areas | Head and Neck | 2010 |  |  | X |  |  |  |  |  |
| Mohammed | Comparing Characteristics of Endometrial Cancer in Women of South Asian and White Ethnicity in England | South Asian women are diagnosed with endometrial cancer at a young age than white women. | Cancer | 2021 | X |  |  | X |  |  |  |  |
| Moller | Short-term breast cancer survival in relation to ethnicity, stage, grade and receptor status: national cohort study in England | Black women experience excess short term mortality compared to white women. | British Journal of Cancer | 2016 |  |  |  | X |  |  |  |  |
| Moller | Colorectal cancer survival in socioeconomic groups in England: variation is mainly in the short term after diagnosis | Survival is worse in lower SES groups and the differences are driven by death close to diagnosis | European Journal of Cancer | 2012 |  |  | X |  |  |  |  |  |
| Moon | Nonadherence to tamoxifen in breast cancer survivors: A 12 month longitudinal analysis | Women who were non-adherent were more likely to be from a minority ethnic group | Health Psychology | 2019 |  |  |  | X |  |  |  |  |
| Morris | Thirty-day postoperative mortality after colorectal cancer surgery in England | Postoperative mortality increased with increasing age. Postoperative mortality increased with increasing comorbidity. Postoperative mortality increased with decreasing SES | Gut | 2011 | X | X | X |  |  |  |  |  |
| Morris | Do pre-diagnosis primary care consultation patterns explain deprivation-specific differences in net survival among women with breast cancer? An examination of individually-linked data from the UK West Midlands cancer registry, national screening programme and Clinical Practice Research Datalink | Women in more deprived categories experienced significantly longer periods between cancer diagnosis and first surgery. Survival was lower in women from more deprived categories | BMC Cancer | 2017 |  |  | X |  |  |  |  |  |
| Morris | What might explain deprivation-specific differences in the excess hazard of breast cancer death amongst screen-detected women? Analysis of patients diagnosed in the West Midlands region of England from 1989 to 2011 | A SES gradient in mortality exists even for screen-detected women. | Oncotarget | 2016 |  |  | X |  |  |  |  |  |
| Morris | Ethnicity, deprivation and screening: survival from breast cancer among screening-eligible women in the West Midlands diagnosed from 1989 to 2011 | No differences in survival by ethnicity. Increasing deprivation associated with worse survival | British Journal of Cancer | 2015 |  |  | X | X |  |  |  |  |
| Morris | Socioeconomic variation in uptake of colonoscopy following a positive faecal occult blood test result: a retrospective analysis of the NHS Bowel Cancer Screening Programme | Variation in colonoscopy uptake by deprivation group is small | British Journal of Cancer | 2012 | X |  | X | X |  |  |  | X |
| Moss | The Effect of Country of Birth on the Pattern of Disease and Survival from Cervical Cancer | Country of birth is not associated with survival | Journal of Lower Genital Tract Disease | 2016 |  |  |  | X |  |  |  |  |
| Moss | Surgical trends, outcomes and disparities in minimal invasive surgery for patients with endometrial cancer in England: a retrospective cohort study | Minimally invasive surgery rates are lower in Black patients compared to white and Asian patients. There was significant variation in minimally invasive surgery by region. Minimally invasive surgery rates were significantly lower in patients from the lowest socioeconomic group compared with patients from the highest group | BMJ Open | 2020 |  |  | X | X |  |  |  |  |
| Moss | Performance measures in three rounds of the English bowel cancer screening pilot | Non-white ethnic background was associated with low bowel cancer screening and colonoscopy uptake. Deprivation was associated with low screening and colonoscopy uptake. Uptake was lower in men | Gut | 2012 |  |  | X | X |  |  |  | X |
| Muquit | Socio-economic characteristics of patients with glioblastoma multiforme | Increasing incidence of glioblastoma multiforme was associated with increasing wage, less unemployment, socioeconomic affluence, lower population density and greater ownership of cars | Journal of Neuro-Oncology | 2015 |  |  | X |  |  |  |  |  |
| Murage | Geographical access to GPs and modes of cancer diagnosis in England: a cross-sectional study | Longer travel was associated with increased risk of diagnosis via emergency and diagnosis by death certificate only (DCO), but decreased risk of diagnosis via screening and two week wait. Patients travelling over 30 minutes had the highest risk of a DCO diagnosis | Family Practice | 2019 |  |  |  |  | X |  |  |  |
| Murage | Geographical disparities in access to cancer management and treatment services in England | Longer average travel times were associated with worse survival | Health & Place | 2016 |  |  |  |  | X |  |  |  |
| Murage | Impact of travel time and rurality on presentation and outcomes of symptomatic colorectal cancer: a cross-sectional cohort study in primary care | Living in a rural area, and travelling farther to a GP in urban areas, may reduce the likelihood of emergency admissions and poor survival | British Journal of General Practice | 2017 |  |  |  |  | X |  |  |  |
| *Murchie* | *Is place or person more important in determining higher rural cancer mortality? A data-linkage study to compare individual versus area-based measures of deprivation* | *Those living >60 minutes from hospital received timely treatment more often than those living <15 minutes. Those living >60 minutes from hospital had a higher one year mortality than those living <15 minutes away. Adjusting for SES did not affect these results* | *International Journal of Population Data Science* | *2021* |  |  | *X* |  | *X* |  |  |  |
| Murchie | Comparing personal risk, melanoma knowledge and protective behaviour in people with and without melanoma: a postal survey to explore educational needs in northeast Scotland | Older age was associated with higher levels of predictive behaviour and younger age with greater concern and knowledge. Higher educational status was associated with increased concern and greater knowledge. Women had greater awareness of risk factors than men | Journal of Cancer Education | 2011 | X |  | X |  |  |  |  | X |
| Myriokefalitaki | Cervical cancer still presents symptomatically 20 years after the introduction of a structured national screening programme | Women who presented symptomatically were older than asymptomatic women. Women at risk of social isolation were more likely to present with symptoms, as an emergency and with advanced disease | Cytopathy | 2016 | X | X |  |  |  |  | X |  |
| *Nartey* | *Is the English Cancer Patient Experience Survey representative? A comparative analysis with the National Lung Cancer Audit* | *CPES is under-represented older people. CPES under-represented people with greater comorbidity or worse performance status. CPES is reasonably ethnically representative. CPES under-represented people who presented as an emergency. CPES under-represented people from lower SES groups. CPES is reasonably representative of sex.* | *Lung cancer* | *2020* | *X* | *X* | *X* | *X* |  |  | *X* |  |
| Nartey | Using patient experiences to evaluate care and expectations in lung cancer: analysis of the English Cancer Patient Experience Survey linked with the national cancer registry | Older people reported more positive experiences of care. People from non-White groups reported worse experience of care. People living in more deprived areas reported more positive experiences of care. Females reported worse experience of care compared to males | Supportive Care in Cancer | 2022 | X |  | X | X |  |  |  | X |
| Nderitu | Prostate-specific antigen testing in inner London general practices: are those at higher risk most likely to get tested? | Use of PSA testing was associated with increasing age. Use of PSA testing was associated with higher BMI and cardiovascular comorbidity. PSA testing was higher in Black men compared to White. Increasing social deprivation was associated with decreasing use of PSA testing | BMJ Open | 2016 | X | X | X | X |  |  |  |  |
| Nelson | The Effect of Rural Residence on Cancer‐Related Self‐Efficacy With UK Cancer Survivors Following Treatment | There was increased cancer-related self-efficacy in rural areas. | Journal of Rural Health | 2022 |  |  |  |  | X |  |  |  |
| Nelson | Health-Promoting Behaviours following Primary Treatment for Cancer: A Rural-Urban Comparison from a Cross-Sectional Study | People living in rural areas were found to have high health responsibility, nutrition, spiritual growth and inter-personal relationships | Current Oncology | 2023 |  |  |  |  | X |  |  |  |
| Nelson | Experiences of cervical screening participation and non-participation in women from minority ethnic populations in Scotland. | Experiences of cervical screening participation vary in some aspects for ethnic minorities | Health Expectations | 2021 |  |  |  | X |  |  |  |  |
| Nesargikar | Consenting for pelvic nerve injury in colorectal surgery: need to address age and gender bias | Younger people were more likely to be consented than older people. Men were more likely to be consented than women | Annals of the Royal College of Surgeons of England | 2010 | X |  |  |  |  |  |  | X |
| Ng | Overall survival of black and white men with metastatic castration-resistant prostate cancer (mCRPC): a 20-year retrospective analysis in the largest healthcare trust in England | Incidence was higher in Black men compared to White. Survival in the Black population was better in those who received hormone-based treatment | Prostate Cancer and Prostatic Diseases | 2021 |  |  |  | X |  |  |  |  |
| Nicholson | Quality of care in rectal cancer surgery. Exploring influencing factors in the West of Scotland | No variation in use of operation type via SES. No variation in operation type by sex | Colorectal Disease | 2012 | X |  | X |  |  |  |  | X |
| Niksic | Is cancer survival associated with cancer symptom awareness and barriers to seeking medical help in England? An ecological study | Substantial variation in cancer awareness and barriers to help seeking across the country. Low cancer awareness is associated with low cancer survival. Barriers to help seeking was not associated with survival except from breast cancer. Low cancer awareness is associated with greater deprivation | British Journal of Cancer | 2016 |  |  | X |  | X |  |  |  |
| Niksic | Ethnic differences in cancer symptom awareness and barriers to seeking medical help in England | Awareness of cancer symptoms is lower in minority ethnic groups, who are additionally more likely to report barriers to help-seeking than white populations. The barriers varied between ethnic groups. | British Journal of Cancer | 2016 |  |  |  | X |  |  |  |  |
| Njoku | Impact of socio-economic deprivation on endometrial cancer survival in the North West of England: a prospective database analysis | Women in the lowest SES groups were more likely to develop fatal recurrence but there were no differences in overall survival by SES group | British Journal of Obstetrics and Gynaecology | 2021 |  |  | X |  |  |  |  |  |
| Njoku | Socioeconomic variation in survival from childhood leukaemia in northern England, 1968-2010 | Survival was lowest in the most deprived groups and this was not explained by access to health care | British Journal of Cancer | 2013 |  |  | X |  |  |  |  |  |
| Nnoaham | Do geodemographic typologies explain variations in uptake in colorectal cancer screening? An assessment using routine screening data in the south of England | Uptake was lower in younger people. Uptake was lower in people in lower SES groups. Geodemographic segmentation explained more of the variation than SES alone. Geodemographic types best predicting response in areas were ethnic mix, and a higher proportion of single pensioner households renting council properties. Uptake was lower in men | Journal of Public Health | 2010 | X |  | X | X |  |  |  | X |
| Norris | Socioeconomic Inequalities in Novel NSCLC Treatments During the Era of Tumour Biomarker-Guided Therapy: A Population-Based Cohort Study in a Publicly Funded Health Care System | Patients living in the most deprived areas were significantly less likely to receive novel treatments than those in more affluent areas | Journal of Thoracic Oncology | 2023 |  |  | X |  |  |  |  |  |
| Nur | Inequalities in non-small cell lung cancer treatment and mortality | Older deprived patients and those who did not receive surgery had much higher odds of death X year after being diagnosed with cancer. Significant geographical variation in use of surgery and X-year mortality | Journal of Epidemiology and Community Health | 2015 | X |  | X |  | X |  |  |  |
| Nur | The impact of age at diagnosis on socioeconomic inequalities in adult cancer survival in England | The magnitude and pattern of the age specific socioeconomic inequalities in survival was different for breast, colon and lung but socioeconomically deprived groups had consistently works outcomes than more affluent groups | Cancer Epidemiology | 2015 | X |  | X |  |  |  |  |  |
| O'Connor | Infection-related mortality in children with acute lymphoblastic leukemia: an analysis of infectious deaths on UKALL2003 | Children with Down syndrome were at significantly higher risk for infection-related mortality during treatment. There was a trend for girls of a higher risk of infection-related mortality during treatment | Blood | 2014 |  | X |  |  |  |  |  | X |
| O'Dowd | What characteristics of primary care and patients are associated with early death in patients with lung cancer in the UK? | Increasing age was associated with early death. Early death was associated with living in a rural area. Smoking was associated with early death. Early death most common in GP practices who requested more chest X-rays but no relationship to level of incidence of lung cancer. Increasing deprivation associated with increasing early mortality | Thorax | 2015 | X |  | X |  | X |  |  | X |
| Offor | Is there a socioeconomic variation in survival from renal tumours in children and young people resident in northern England (1968-2012)? | No variation in survival by SES | Cancer Epidemiology | 2017 |  |  | X |  |  |  |  |  |
| Oliphant | The changing association between socioeconomic circumstances and the incidence of colorectal cancer: a population-based study | Deprivation was associated with higher incidence rates of cancer in males but not females | British Journal of Cancer | 2011 |  |  | X |  |  |  |  |  |
| Oliphant | The impact of surgical specialisation on survival following elective colon cancer surgery | Survival was better in people treated by specialist surgeons due to the higher postoperative mortality rates of non-specialists | International Journal of Colorectal Disease | 2014 |  |  |  |  |  |  | X |  |
| Oliphant | Deprivation and colorectal cancer surgery: longer-term survival inequalities are due to differential postoperative mortality between socioeconomic groups | People in the most deprived socioeconomic groups had the worst survival and this was explained by higher rates of post-operative mortality | Annals of Surgical Oncology | 2013 |  |  | X |  |  |  |  |  |
| Orchard | Demographic trends in the incidence of malignant appendiceal tumours in England between 1995 and 2016: Population-based analysis | The biggest increases in incidence over time were seen in young people. No differences in incidence trends by SES. No differences in incidence trends by sex | BJS Open | 2022 | X |  | X |  |  |  |  | X |
| *O'Reilly* | *Using record linkage to monitor equity and variation in screening programmes* | *Screening data can be linked to census data for increasingly powerful analyses* | *BMC Medical Research Methodology* | *2012* |  |  | *X* |  |  |  |  |  |
| O'Reilly | Uptake of breast screening is influenced by current religion and religion of upbringing | Uptake was lower in women with no current religious affiliation. There were modest differences across different religious affiliations. Amongst those with no current religious affiliation, religion of upbringing positively influence uptake rates | Journal of Religion and Health | 2013 |  |  |  |  |  |  | X |  |
| Osborn | Access to cancer screening in people with learning disabilities in the UK: cohort study in the health improvement network, a primary care research database | People with learning disabilities are significantly less likely to participate in screening | PloS One | 2012 |  | X |  |  |  |  |  |  |
| Padmanabhan | Ethnic variation in colorectal cancer risk following a positive faecal occult blood test in an English bowel cancer screening programme centre | The risk of cancer (and adenoma) is higher in non-South Asians than in South Asians | European Journal of Gastroenterology and Hepatology | 2015 |  |  |  | X |  |  |  |  |
| Parry | Determinants of variation in radical local treatment for men with high-risk localised or locally advanced prostate cancer in England | Radical local treatment was more common in younger men. Radical local treatment was more common in men with fewer comorbidities. Radical local treatment was more common in men from a non-Black ethnic group. There was significant variation between hospitals in the use of radical local treatment. Use of radical local treatment increased with increasing SES | Prostate Cancer and Prostatic Diseases | 2021 | X | X | X | X | X |  |  |  |
| Patel | Awareness of and attitudes towards cervical cancer prevention among migrant Eastern European women in England | Women migrants from Eastern Europe did not have full knowledge of the screening system and did not fully participate in screening | Journal of Medical Screening | 2019 |  |  |  | X |  |  |  |  |
| Patel-Kerai | The psychosocial experiences of breast cancer amongst Black, South Asian and White survivors: do differences exist between ethnic groups? | South Asians reported higher levels of anxiety and depression, poorer quality of life and held higher levels of internal and fatalistic beliefs about cancer compared to White women. Black and South Asian women reported higher levels of body image concerns and held stronger beliefs that God was in control of their cancer than white women. South Asian women turned to religion as a source of support more than Black and White women | Psycho-Oncology | 2017 |  |  |  | X |  |  |  |  |
| Paterson | Deprivation and access to treatment for colorectal cancer in Southeast Scotland 2003-2009 | Treatment rates were higher in the least deprived groups. Deprivation was not associated with treatment delay or advanced stage of disease at presentation | Colorectal Disease | 2014 |  |  | X |  |  |  |  |  |
| Perez-Cornago | Prospective investigation of risk factors for prostate cancer in the UK Biobank cohort study | High BMI, smoking, having diabetes, never having had children or sexual intercourse were associated with a lower risk. Family history of prostate cancer, having an enlarged prostate or having a prostate specific antigen test were associated with a higher risk. Risk was highest in people of Black ethnicity and Asian ethnicity lowest | British Journal of Cancer | 2017 |  |  |  | X |  |  |  |  |
| Pethick | Inpatient admissions and outpatient appointments in the first year post cancer diagnosis: A population based study from England | Younger patients tended to longer stays | Cancer Epidemiology | 2021 | X |  |  |  |  |  |  |  |
| *Pham* | *Predictors of Postal or Online Response Mode and Associations With Patient Experience and Satisfaction in the English Cancer Patient Experience Survey* | *Younger people were more likely than older to respond online rather than by post. Non- White responders to were more likely to respond online (rather than by post) than white responders. People from more affluent areas were more likely to respond online rather than by post. Males more likely than females to respond online rather than by post. Online responders were more likely to report better experience of care* | *Journal of Medical Internet Research* | *2019* | *X* |  | *X* | *X* |  |  |  | *X* |
| Philips | Socioeconomic differences impact overall survival in advanced ovarian cancer (AOC) prior to achievement of standard therapy | Surgery rates were higher in more affluent groups and people refusing any treatment were in the lowest three SES groups. SES was not a predictor of survival | Archives of Gynaecology and Obstetrics | 2019 |  |  | X |  |  |  |  |  |
| Pi | Body composition and risk of liver cancer: a population-based prospective cohort study on gender difference | Males with the highest body fat at greatest risk. U-shaped relationship with body fat for females | Frontiers in nutrition | 2023 |  |  |  |  |  |  |  | X |
| Pinder | Minority ethnicity patient satisfaction and experience: results of the National Cancer Patient Experience Survey in England | People from ethnic minority groups reported lower satisfaction and less positive experiences of care as well as lower confidence and less understanding of health care professionals | BMJ Open | 2016 |  |  |  | X |  |  |  |  |
| Pirie | Lung cancer in never smokers in the UK Million Women Study | Incidence was increased in people with asthma requiring treatment. Incidence was increased in non-White people compared to White. Incidence was increased in people of taller stature. 31 other risks factors were not significant | International Journal of Cancer | 2016 |  | X |  | X |  |  |  |  |
| Pollock | Evidence of decreased HPV vaccine acceptance in Polish communities within Scotland | Lower uptake from girls from Polish families | Vaccine | 2019 |  |  |  | X |  |  |  |  |
| Pompili | Factors influencing patient satisfaction after treatments for early-stage non-small cell lung cancer | Patient living in more deprived areas were more satisfied with care | Journal of Cancer Research and Clinical Oncology | 2022 |  |  | X |  |  |  |  |  |
| Price | Comparison of breast and bowel cancer screening uptake patterns in a common cohort of South Asian women in England | South Asian women were significantly less likely to undertake both breast and bowel cancer screening compared to non-Asians. | BMC Health Services Research | 2010 |  |  |  | X |  |  |  |  |
| Prurkayastha | Trends of oral cavity, oropharyngeal and laryngeal cancer incidence in Scotland (1975-2012) - A socioeconomic perspective | People from the most deprived areas have the highest incidence of these cancers. Men had a higher incidence of oropharyngeal and laryngeal cancer than females | Oral Oncology | 2016 |  |  | X |  |  |  |  | X |
| Pulte | Survival for patients with chronic leukemias in the US and Britain: Age-related disparities and changes in the early 21st century | Survival is improving but older people consistently have worse outcomes than younger people | European Journal of Haematology | 2015 | X |  |  |  |  |  |  |  |
| Quaresma | Variation in colon cancer survival for patients living and receiving care in London, 2006-2013: does where you live matter? | Geographical differences in survival disappeared after adjusting for hospital of treatment. However, poorer survival at some hospitals we due to higher proportions of people presenting as an emergency. | Journal of Epidemiology and Community Health | 2022 |  |  |  |  | X |  |  |  |
| Quyn | Uptake trends in the Scottish Bowel Screening Programme and the influences of age, sex, and deprivation | Screening uptake increased with age. Screening uptake was lower in people living in more deprived areas but the number needed to screen to find significant disease was lower in this group too. Uptake was lower in men than women but the number of men needed to screen to find significant disease was lower | Journal of Medical Screening | 2018 | X |  | X |  |  |  |  | X |
| Rachet | Socioeconomic inequalities in cancer survival in England after the NHS cancer plan | Survival improved over time but a deprivation gap persisted. The majority of this gap is a result of deaths close to diagnosis | British Journal of Cancer | 2010 |  |  | X |  |  |  |  |  |
| Radu | Awareness of ovarian cancer symptoms and risk factors in a young ethnically diverse British population | Help seeking behaviour was higher in Black and Asian ethnicities. Males had lower symptom recall than females | Cancer Medicine | 2023 |  |  |  | X |  |  |  | X |
| Radwan | Social deprivation in patients requiring pelvic exenterative surgery | SES is significantly associated with postoperative length of stay and survival in people undergoing pelvic exenteration | Colorectal Disease | 2016 |  |  | X |  |  |  |  |  |
| Rafiq | Socioeconomic deprivation and regional variation in Hodgkin's lymphoma incidence in the UK: a population-based cohort study of 10 million individuals | Incidence of Hodgkin’s disease was higher in people residing in more affluent areas | BMJ Open | 2019 |  |  | X |  |  |  |  |  |
| Raine | Social variations in access to hospital care for patients with colorectal, breast, and lung cancer between 1999 and 2006: retrospective analysis of hospital episode statistics | Older people were more likely to be admitted as emergencies. Older people were less likely to receive breast conserving surgery and lung cancer resection. People living in deprived areas were more likely to be admitted as an emergency and receive preferred procedures. Men were less likely than women to undergo anterior resection for rectal cancer and lung cancer resection | British Medical Journal | 2010 | X |  | X |  |  |  |  | X |
| Rajan | Late presentation and management of South Asian breast cancer patients in West Yorkshire, United Kingdom | South Asian women were less likely to present via screening and have advanced disease than other ethnicities. | Asian Pacific Journal of Cancer Prevention | 2011 |  |  |  | X |  |  |  |  |
| Ramsay | Time trends in socioeconomic inequalities in cancer mortality: results from a 35 year prospective study in British men | Cancer mortality in men who work in manual jobs versus no-manual is significantly higher. This difference remains unchanged over time | BMC Cancer | 2014 |  |  | X |  |  |  |  |  |
| Ratneswaren | The survival of patients with high grade glioma from different ethnic groups in South East England | Receipt of treatment was consistent across all ethnic groups. Indian and Other ethnic groups had better survival, and the non-known group worse survival, than the White group. Survival in the Black and White groups was similar | Journal of Neuro-Oncology | 2014 |  |  |  | X |  |  |  |  |
| Ravaghi | Area-level deprivation and oral cancer in England 2012-2016 | Incidence and mortality higher in Asian groups. Incidence and mortality increased with alcohol consumption. Incidence and mortality higher in more deprived areas | Cancer Epidemiology | 2020 |  |  | X | X |  |  |  |  |
| Rebolj | Concurrent participation in screening for cervical, breast, and bowel cancer in England | Women from general practices from areas with less deprivation were more likely to participate in all screening programmes. Women from general practices with more patients who are carers, have chronic illnesses themselves and with more satisfaction of services were more likely to participate in screening | Journal of Medical Screening | 2020 |  |  | X |  |  |  | X |  |
| Renshaw | Estimating attendance for breast cancer screening in ethnic groups in London | There was little difference in attendance by age. Uptake was low in Black populations compared to other ethnic groups. Uptake was lower in lower SES groups | BMC Public Health | 2010 | X |  | X | X |  |  |  |  |
| Renshaw | Trends in the incidence and survival of multiple myeloma in South East England 1985-2004 | Trend for better survival in more affluent areas | BMC Cancer | 2010 | X |  | X |  |  |  |  | X |
| Rewhorn | Rare male cancers: Effect of social deprivation on a cohort of penile cancer patients | Incidence, grade and stage of disease at diagnosis were all greater in men residing in lower SES areas | Journal of Clinical Urology | 2021 |  |  | X |  |  |  |  |  |
| Riaz | Lung cancer incidence and survival in England: an analysis by socioeconomic deprivation and urbanization | The difference in incidence and survival between rural and urban areas is largely explained by SES | Journal of Thoracic Oncology | 2011 | X |  | X |  | X |  |  |  |
| Riaz | Recent trends in resection rates among non-small cell lung cancer patients in England | Increasing age was associated with a reduction in pneumonectomy and sleeve resection compared with lobectomy and a slight increase in wedge resection. Surgery rates increased across all SES groups over time but differences between groups remained | Thorax | 2012 | X |  | X |  |  |  |  | X |
| Rich | How do patient and hospital features influence outcomes in small-cell lung cancer in England? | Increasing age was associated with a lower likelihood of receiving chemotherapy. Worsening performance status and increasing comorbidity associated with a reduced likelihood of receiving chemotherapy. Wide variation in chemotherapy use across hospitals | British Journal of Cancer | 2011 | X | X |  |  | X |  |  |  |
| Rich | Inequalities in outcomes for non-small cell lung cancer: the influence of clinical characteristics and features of the local lung cancer service | The minority of people who first present to a hospital that is a thoracic surgical centre are more likely to have surgery and so receive the benefit this confers | Thorax | 2011 |  |  |  |  | X |  |  |  |
| Rich | Non-small cell lung cancer in young adults: presentation and survival in the English National Lung Cancer Audit | Young people are more likely to have treatment and survive longer than older people | QJM | 2015 | X |  |  |  |  |  |  |  |
| Robb | Flexible sigmoidoscopy screening for colorectal cancer: uptake in a population-based pilot programme | Uptake was higher in people living in more affluent areas. Uptake did not differ by gender | Journal of Medical Screening | 2010 |  |  | X |  |  |  |  | X |
| Robertson | Explaining the effects of socio-economic deprivation on survival in a national prospective cohort study of 1909 patients with head and neck cancers | After adjustment for other factors (age, tumour site, smoking status, alcohol use, tumour differentiation) the SES differences in survival disappeared | Cancer Epidemiology | 2010 |  |  | X |  |  |  |  |  |
| Ross | Disability as a predictor of breast cancer screening uptake: A population-based study of 57,328 women | Women with more than one chronic disability were less likely to participate in screening | Journal of Medical Screening | 2020 |  | X |  |  |  |  |  |  |
| Ross | Does poor mental health explain socio-demographic gradients in breast cancer screening uptake? A population-based study | People with poor mental health were less likely to participate in screening | European Journal of Public Health | 2020 |  | X |  |  |  |  |  |  |
| Ross | Disparities in Breast Cancer Screening Uptake for Women With Mental Illness in the United Kingdom | Screening uptake was less likely in women with mental illness and particularly low for women prescribed antipsychotics, anxiolytics and hypnotics | American Journal of Preventative Medicine | 2021 |  | X |  |  |  |  |  |  |
| Rutherford | The impact of eliminating age inequalities in stage at diagnosis on breast cancer survival for older women | If the differences in stage at presentation by age were eliminated then survival could be improved in older women | British Journal of Cancer | 2015 | X |  |  |  |  |  |  |  |
| Rutherford | Understanding the impact of socioeconomic differences in breast cancer survival in England and Wales: avoidable deaths and potential gain in expectation of life | Socioeconomic differences in relative survival have an impact on life expectancy for patients and result in higher early mortality for more deprived patients. However, differences in general survival across socioeconomic groups explain a larger proportion of the deprivation gap in life expectancy for breast cancer patients. | Cancer Epidemiology | 2015 |  |  | X |  |  |  |  |  |
| Rutherford | How much of the deprivation gap in cancer survival can be explained by variation in stage at diagnosis: an example from breast cancer in the East of England | Affluent women are less likely to be diagnosed with advanced disease. Eliminating SES difference in stage at diagnosis would reduce the survival gap | International Journal of Cancer | 2013 |  |  | X |  |  |  |  |  |
| Ryan | Mortality due to cardiovascular disease, respiratory disease, and cancer in adults with cerebral palsy | Adults with cerebral palsy did not have a higher risk of cancer mortality than those without | Developmental Medicine and Child Neurology | 2019 |  | X |  |  |  |  |  |  |
| Rylands | Outcomes by area of residence deprivation in a cohort of oral cancer patients: Survival, health-related quality of life, and place of death | Increasing deprivation associated with worse quality of life, particularly socioemotional function | Oral Oncology | 2016 |  |  | X |  |  |  |  |  |
| Rylands | Influence of deprivation on health-related quality of life of patients with cancer of the head and neck in Merseyside and Cheshire | People living in more deprived areas had worse survival and quality of life in regard to social-emotional functioning and overall but in physical oral function | British Journal of Oral and Maxillofacial Surgery | 2016 |  |  | X |  |  |  |  |  |
| Sacks | Uptake of the HPV vaccination programme in England: a cross-sectional survey of young women attending sexual health services | Offer and uptake rates of vaccination lower in non-White ethnic groups. Offer and uptake rates of vaccination lower in London. Offer and uptake rates of vaccination lower in those with previous sexually transmitted infections. Offer and uptake rates of vaccination lower in people not in education, training or employment | Sexually Transmitted Infections | 2014 |  |  | X | X | X |  |  |  |
| Saito | Exploring socioeconomic differences in surgery and in time to elective surgery for colon cancer in England: Population-based study | Time to surgery did not differ by SES group but emergency surgery was higher in people from more deprived areas | Cancer Epidemiology | 2021 |  |  | X |  |  |  |  |  |
| Samy | Variation in incidence and survival by ethnicity for patients with myeloma in England (2002-2008) | Incidence was higher in Black groups compared to South Asian or White. Survival was better in Black and South Asian groups compared to White | Leukaemia and Lymphoma | 2015 |  |  |  | X |  |  |  |  |
| *Saunders* | *Accuracy of routinely recorded ethnic group information compared with self-reported ethnicity: evidence from the English Cancer Patient Experience survey* | *There are differences between self-reported and routinely recorded ethnicity* | *BMJ Open* | *2013* |  |  |  | *X* |  |  |  |  |
| Saunders | Inequalities in reported cancer patient experience by socio-demographic characteristic and cancer site: evidence from respondents to the English Cancer Patient Experience Survey | Younger and very old, people reported worse experience. Ethnic minority patients reported worse experience. Women consistently reported worse experiences across questions | European Journal of Cancer Care | 2015 | X |  |  | X |  |  |  | X |
| Saunders | Long-term conditions among sexual minority adults in England: evidence from a cross-sectional analysis of responses to the English GP Patient Survey | Although there were differences in the incidence of other conditions in sexual minority adults there was no difference in cancer incidence | BJGP Open | 2021 |  |  |  |  |  | X |  |  |
| Saunders | Cervical screening attendance and cervical cancer risk among women who have sex with women | Women who have sex with women and women and men were less likely to participate in cervical screening. There were no differences in bowel screening. In breast screening attendance was lower in women who have sex with men and women | Journal of Medical Screening | 2021 |  |  |  |  |  | X |  |  |
| Saunders | Associations Between Sexual Orientation and Overall and Site-Specific Diagnosis of Cancer: Evidence From Two National Patient Surveys in England | Incidence of cancer by site does not vary substantially by sexual orientation with the exception of some HPV- and HIV associated cancers | Journal of Clinical Oncology | 2017 |  |  |  |  |  | X |  |  |
| Sayeed | Childhood cancer incidence by ethnic group in England, 2001-2007: a descriptive epidemiological study | Black children and Pakistani children have an increased risk of all cancers. South Asian children have increased risk of Lymphoma and leukaemia, but decreased risk of CNS cancers. Black children have increased risk of lymphoma | BMC Cancer | 2017 |  |  |  | X |  |  |  |  |
| Sayeed | Childhood cancer incidence in British Indians & Whites in Leicester, 1996-2008 | All cancer incidence is higher in Indian children than white children largely due to a higher incidence of central nervous system and haematological malignancies | PloS One | 2013 |  |  |  | X |  |  |  |  |
| Sekhon Inderjit Singh | Ethnic disparities in the uptake of colorectal cancer screening: An analysis of the West London population | All ethnic minority patients except Chinese patients have significantly lower compliance with colorectal cancer screening. | Colorectal Disease | 2021 |  |  |  | X |  |  |  |  |
| Shafique | Socio-economic inequalities in survival of patients with prostate cancer: role of age and Gleason grade at diagnosis | Increasing deprivation was associated with an increased risk of death | PloS One | 2013 |  |  | X |  |  |  |  |  |
| Shafique | The impact of socio-economic circumstances on overall and grade-specific prostate cancer incidence: a population-based study | Prostate cancer incidence is increasing and an increase in low grade disease in affluent men may suggest prostate-specific antigen testing is partly responsible | British Journal of Cancer | 2012 |  |  | X |  |  |  |  |  |
| Shah | Place of death and hospital care for children who died of cancer in England, 1999-2006 | Asian and Black children were more likely to die in hospital than at home. Children from more deprived areas were more likely to die at home | European Journal of Cancer | 2011 |  |  | X | X |  |  |  |  |
| Shah | Recruitment of childhood leukaemia patients to clinical trials in Great Britain during 1980-2007: variation by birth weight, congenital malformation, socioeconomic status and ethnicity | Although overall recruitment to trials is high it is lower in children with lower birth weight, a congenital malformation, lower SES and in ethnic minority groups | Archives of Disease in Childhood | 2014 |  | X |  | X |  |  |  |  |
| Shah | Leukaemia survival trends in children with Down's syndrome in Great Britain, 1971-2000: a population-based study | Survival has improved for all children but those with Down syndrome have poorer survival than those without | Journal of Epidemiology and Community Health | 2011 |  | X |  |  |  |  |  |  |
| Sharp | Risk of several cancers is higher in urban areas after adjusting for socioeconomic status. Results from a two-country population-based study of 18 common cancers | Risks of breast, cervical, kidney and brain cancer were significantly higher in females in urban areas. Prostate cancer risk was higher in rural areas. Other cancers showed no significant urban–rural differences. | Journal of Urban Health | 2014 |  |  |  |  | X |  |  |  |
| Sharpe | Policy for home or hospice as the preferred place of death from cancer: Scottish Health and Ethnicity Linkage Study population cohort shows challenges across all ethnic groups in Scotland | No differences in place of death by ethnic group. More affluent people are less likely to die in hospital | BMJ Supportive & Palliative Care | 2015 |  |  | X | X |  |  |  |  |
| Sharpe | Socioeconomic inequalities in incidence of lung and upper aero-digestive tract cancer by age, tumour subtype and sex: a population-based study in Scotland (2000-2007) | Socioeconomic inequality varies by age and sex | Cancer Epidemiology | 2012 |  |  | X |  |  |  |  |  |
| Sharpe | Association between socioeconomic factors and cancer risk: a population cohort study in Scotland (1991-2006) | Economic inactivity was associated with increased incidence. No or non-degree qualification (versus degree) was associated with increased lung risk; likewise for upper aero tract cancers (females only). No car access (versus ownership) was associated with increased risk. Renting (versus home ownership) was associated with increased lung cancer risk, UADT cancer risk (males only) and all cancer risk (females only). Elevated risk was associated with no education and living in deprived areas | PloS One | 2014 |  |  | X |  |  |  |  |  |
| Sheringham | Variations in GPs' decisions to investigate suspected lung cancer: a factorial experiment using multimedia vignettes | GPs were less likely to investigate older rather than younger people. GPs were less likely to investigate Black patients than White | BMJ Quality & Safety | 2017 | X |  |  | X |  |  |  |  |
| Shirley | Incidence of breast and gynaecological cancers by ethnic group in England, 2001-2007: a descriptive study | South Asian and Black patients have lower rates of breast, ovarian and cervical cancer than white patients. Chinese patients had lower rates of breast and cervical cancer, and Black patients had higher rates of endometrial cancer than white patients. The study additionally found substantial intra-ethnic differences, particularly amongst South Asian patients. | BMC Cancer | 2014 |  |  |  | X |  |  |  |  |
| Shirley | Incidence of haematological malignancies by ethnic group in England, 2001-7 | White patients have the highest incidence rates for most subtypes of haematological malignancies. However, Black patients had over double the incidence of plasma cell and mature T-cell neoplasms than other ethnic groups. Incidence varied within ethnic subgroups, e.g. Indian, Pakistani, and Bangladeshi patients experienced significantly different incidence of Hodgkin’s lymphoma. | British Journal of Haematology | 2013 |  |  |  | X |  |  |  |  |
| Shiue | Social correlates of total cancer in adults and the very old: UK Understanding Society Cohort, 2009-2010 | In middle aged and young people being female was associated with more cancer. Incidence was greater in people who were obese/overweight. Incidence was greater in people who were born outside the UK | Public Health | 2014 | X |  |  |  |  |  |  | X |
| Smith | Investigating the inequalities in route to diagnosis amongst patients with diffuse large B-cell or follicular lymphoma in England. | Underlying comorbidity is associated with diagnostic delay | British Journal of Cancer | 2021 |  | X |  |  | X |  |  |  |
| Smith | Impact of age and socioeconomic status on treatment and survival from aggressive lymphoma: a UK population-based study of diffuse large B-cell lymphoma | No impact of age on outcome. Worse performance status associated with worse survival. No impact of SES on survival | Cancer Epidemiology | 2015 | X |  | X |  |  |  |  |  |
| Smith | Determinants of survival in patients with chronic myeloid leukaemia treated in the new era of oral therapy: findings from a UK population-based patient cohort | People living in deprived areas have poor survival. Survival was lower in people from lower SES areas. Survival outcomes similar for men and women | BMJ Open | 2014 |  |  | X |  |  |  |  |  |
| *Smith* | *Comparison of ethnic group classification using naming analysis and routinely collected data: application to cancer incidence trends in children and young people* | *Using different methods of assigning ethnicity can result in different estimates of ethnic variation in cancer incidence* | *BMJ Open* | *2017* |  |  |  | *X* |  |  |  |  |
| Smith | Excess Mortality by Multimorbidity, Socioeconomic, and Healthcare Factors, amongst Patients Diagnosed with Diffuse Large B-Cell or Follicular Lymphoma in England | Deprivation consistently associated with poorer survival | Cancer | 2021 |  |  | X |  |  |  |  |  |
| Smith | Association between multimorbidity and socioeconomic deprivation on short-term mortality among patients with diffuse large B-cell or follicular lymphoma in England: a nationwide cohort study | Multi-morbidity was associated with higher X-year mortality. Increasing deprivation was associated with higher X-year mortality | BMJ Open | 2021 |  | X | X |  |  |  |  |  |
| Smith | Mediating Effects of Diagnostic Route on the Comorbidity Gap in Survival of Patients with Diffuse Large B-Cell or Follicular Lymphoma in England | Those with any comorbidity were more likely to experience diagnostic delay and poorer survival. Those living in more deprived areas were more likely to experience diagnostic delay and poorer survival | Cancers | 2022 |  | X | X |  |  |  |  |  |
| Smith | The associations between objective numeracy and colorectal cancer screening knowledge, attitudes and defensive processing in a deprived community sample | Low numeracy respondents were less knowledgeable about colorectal cancer | Journal of Health Psychology | 2016 |  |  | X |  |  |  |  |  |
| Smith | Inequalities in cancer screening participation: examining differences in perceived benefits and barriers | People with high school education or no formal education reported higher emotional and practical barriers and were less likely to definitely intend to participate in screening | Psycho-Oncology | 2016 |  |  | X |  |  |  |  |  |
| Spencer | Sociodemographic factors predicting mother's cervical screening and daughter's HPV vaccination uptake | There were differences in screening and vaccination uptake by PCT. Deprivation was not associated with routine (12–13-year-olds) vaccination initiation, but girls living in the most deprived quintile were significantly less likely to complete the three vaccine doses. Mother–daughter pairs failing to engage in either screening or vaccination were also more likely to live in deprived areas. | Journal of Epidemiology and Community Health | 2014 |  |  | X |  |  |  |  |  |
| Steele | Effect of gender, age and deprivation on key performance indicators in a FOBT-based colorectal screening programme | Uptake, positivity and cancer detection rates increased with age. Uptake high in women than man. Positivity rates increased with increasing deprivation. Cancer detection rates not affected. PPV decreased with increasing deprivation | Journal of Medical Screening | 2010 | X |  | X |  |  |  |  | X |
| Stringhini | Socioeconomic Status, Structural and Functional Measures of Social Support, and Mortality | Measures of social support were not associated with cancer mortality | American Journal of Epidemiology | 2012 |  |  | X |  |  |  |  |  |
| Sturley | Contrasting socio-economic influences on colorectal cancer incidence and survival in England and Wales | No clear relationship of are of deprivation and incidence. Survival negatively associated with increasing deprivation | Social Science & Medicine | 2023 |  |  | X |  |  |  |  |  |
| Syriopoulou | Estimating the impact of a cancer diagnosis on life expectancy by socio-economic group for a range of cancer types in England | There are wide differences in the impact of cancer on life expectancy across deprivation groups, and for most cancers the most affluent lose less years | British Journal of Cancer | 2017 |  |  | X |  |  |  |  |  |
| Syriopoulou | Understanding the impact of socioeconomic differences in colorectal cancer survival: potential gain in life-years | Major differences in survival between the most affluent and most deprived. If these differences could be eliminated there would be substantial life years gained in people living in the most deprived areas | British Journal of Cancer | 2019 |  |  | X |  |  |  |  |  |
| Taib | Socioeconomic deprivation and the burden of head and neck cancer-Regional variations of incidence and mortality in Merseyside and Cheshire, North West, England | Income deprivation has a strong association with increasing incidence and mortality | Clinical Otolaryngology | 2018 |  |  | X |  |  |  |  |  |
| Tanton | High-Risk Human Papillomavirus (HPV) Infection and Cervical Cancer Prevention in Britain: Evidence of Differential Uptake of Interventions from a Probability Survey | Screening non-attendance was associated with younger and older age. Screening non-attendance and lower uptake of vaccination was associated increasing area-level deprivation. Screening non-attendance was high in Asian/Asian British ethnicities and lower uptake of vaccination with non-White ethnicities | Cancer Epidemiology, Biomarkers & Prevention | 2015 | X |  | X | X |  | X |  |  |
| Tataru | Variation in geographical treatment intensity affects survival of non-small cell lung cancer patients in England | wide geographical variation in the use of curative treatment and a higher frequency of treatment was associated with better survival. Significant variation in treatment by deprivation | Cancer Epidemiology | 2018 |  |  | X |  | X |  |  |  |
| Tataru | Trends in the epidemiology of head and neck cancer in London | Compared with their White counterparts, Bangladeshi females had a higher incidence of oral, laryngeal and thyroid cancers; Chinese males and females had a higher incidence of nasopharyngeal cancer; and Pakistani and Indian females and Indian males also had higher incidence of oral cancer. Deprivation was associated with a significantly higher incidence for larynx (males), oropharynx (males and females) and oral cavity (females) | Clinical Otolaryngology | 2017 |  |  | X | X |  |  |  | X |
| Thomas | Modelling cost-effective strategies for minimising socioeconomic inequalities in colorectal cancer screening outcomes in England | Screening was found to be highly cost-effective but CRC inequalities increased as screening effectiveness improved | Preventive Medicine | 2022 |  |  | X |  |  |  |  |  |
| Trenchard | Ethnic variation in cancer patients' ratings of information provision, communication and overall care | Non-White patients (particularly Asian) were less likely than White patients to receive an understandable explanation of treatment side effects. Among Asian patients, those of Bangladeshi ethnicity were least likely to receive an understandable explanation | Ethnicity and Health | 2016 |  |  |  | X |  |  |  |  |
| Turner | A cancer geography paradox? Poorer cancer outcomes with longer travelling times to healthcare facilities despite prompter diagnosis and treatment: a data-linkage study | Increased travelling time to a cancer treatment centre was associated with increased mortality. But increased travelling times was associated with more timely treatment | British Journal of Cancer | 2017 |  |  |  |  | X |  |  |  |
| Tweed | Socio-economic inequalities in the incidence of four common cancers: a population-based registry study | In breast and prostate cancer there was lower incidence in people living in more deprived areas. In colorectal and lung cancer incidence was higher in more deprived areas | Public Health | 2018 |  |  | X |  |  |  |  |  |
| Underwood | Breast, Prostate, Colorectal, and Lung Cancer Incidence and Risk Factors in Women Who Have Sex with Women and Men Who Have Sex with Men: A Cross-Sectional and Longitudinal Analysis Using UK Biobank | No evidence of an association between sexual history and breast, colorectal, or prostate cancer incidence. Lung cancer incidence was higher for women who have sex with women compared with women who have sex exclusively with men, and men who have sex with men compared with men who have sex exclusively with women; accounting for differences in smoking prevalence in these groups explained this higher incidence. | Cancers | 2023 |  |  |  |  |  | X |  |  |
| Vallance | Socioeconomic differences in selection for liver resection in metastatic colorectal cancer and the impact on survival | Lower rates of liver resection in more deprived patients. Deprived CRC patients with synchronous liver-limited metastases have worse survival than more affluent patients | European Journal of Surgical Oncology | 2018 |  |  | X |  |  |  |  |  |
| Van Laar | Cancer incidence among the south Asian and non-south Asian population under 30 years of age in Yorkshire, UK | Overall cancer incidence was similar for south Asians and non-south Asian but rates of increase were higher in South Asians | British Journal of Cancer | 2010 |  |  |  | X |  |  |  |  |
| Van Laar | Survival trends of cancer amongst the south Asian and non-south Asian population under 30 years of age in Yorkshire, UK | Poorer survival outcomes for south Asians compared to non-south Asian children and young adults with leukaemia and lymphoma, but better outcomes for south Asian children and young adults with other solid tumours | Cancer Epidemiology | 2012 |  |  |  | X |  |  |  |  |
| Von Wagner | Inequalities in participation in an organized national colorectal cancer screening programme: results from the first 2.6 million invitations in England | Screening uptake was lower in more deprived areas. More ethnically diverse areas had lower uptake. Uptake was greater in women than men | International Journal of Epidemiology | 2011 |  |  | X | X |  |  |  | X |
| Walker | Type 2 diabetes, socioeconomic status and risk of cancer in Scotland 2001-2007 | Socioeconomic status was found to have little influence on the association between type 2 diabetes and cancer | Diabetologia | 2013 |  | X | X |  |  |  |  |  |
| Wallace | Identifying patients at risk of emergency admission for colorectal cancer | The risk of emergency presentation increased with age. People with dementia or cardiac, neurologic and liver disease had a higher risk of emergency presentation. People of non-White ethnic background had a higher risk of emergency presentation. People from more deprived areas had a higher risk of emergency presentation. Women had a higher risk of emergency presentation | British Journal of Cancer | 2014 | X | X | X | X |  |  |  |  |
| Wallingford | Regional melanoma incidence in England, 1996-2006: reversal of north-south latitude trends among the young female population | Melanoma incidence is high in young people in northern England | British Journal of Dermatology | 2013 |  |  |  |  | X |  |  |  |
| Walsh | Characteristics of men responding to an invitation to undergo testing for prostate cancer as part of a randomised trial | Little difference in age between responders and non-responders. Responders were slightly more likely to come from urban rather than rural areas. Responders were slightly less deprived than those who did not respond | Trials | 2016 | X |  | X |  | X |  |  |  |
| Walsh | Disparities In Cancer Care And Costs At The End Of Life: Evidence From England's National Health Service | Greater use of emergency inpatient care by people of lower SES leading to greater costs | Health affairs | 2017 |  |  | X |  |  |  |  |  |
| Walters | Geographical variation in cancer survival in England, 1991-2006: an analysis by Cancer Network | There was a wide geographical variation in survival with generally lower estimates in northern England | Journal of Epidemiology and Community Health | 2011 |  |  |  |  | X |  |  |  |
| Wanis | The Influence of Ethnicity on Survival from Malignant Primary Brain Tumours in England: A Population-Based Cohort Study | Patients of an Indian background, Any Other White, Other Ethnic Group, and Unknown/Not Stated Ethnicity Groups having better one-year survival than the White British Group | Cancers | 2023 |  |  |  | X |  |  |  |  |
| Warwick | Variation in colorectal cancer treatment and survival: a cohort study covering the East Anglia region. | Proportion of patients in the most deprived quintile is associated with perioperative death | Colorectal Disease | 2013 |  |  | X |  | X |  |  |  |
| Watt | Primary care and cancer: an analysis of the impact and inequalities of the COVID-19 pandemic on patient pathways | The reduction in the number of 2-week wait referrals and first treatments for all cancer has been largest for those living in poorer areas | BMJ Open | 2022 |  |  | X |  |  |  |  |  |
| Whitaker | Socioeconomic inequalities in colorectal cancer screening uptake: does time perspective play a role? | People in lower SES groups had lower uptake and this was associated with higher perceived barrier to and benefits of screening | Health Psychology | 2011 |  |  | X |  |  |  |  |  |
| Whitaker | Low cancer suspicion following experience of a cancer 'warning sign' | Lower education was associated with lower likelihood of cancer suspicion | European Journal of Cancer | 2015 |  |  | X |  |  |  |  |  |
| White | Geographic variation in the use of lymphadenectomy and external-beam radiotherapy for endometrial cancer: a cross-sectional analysis of population-based data | There was substantial variation by Cancer Alliance in the adjusted proportion of women with endometrial cancer receiving specific treatments | British Journal of Obstetrics and Gynaecology | 2019 |  |  |  |  | X |  |  |  |
| Whynes | Analysis of deaths occurring within the Nottingham trial of faecal occult blood screening for colorectal cancer | Mean age at death was higher for colorectal cancer than for other cancers, except for prostate cancer among men. Increasing levels of deprivation significantly lowered the expected ages at death independently of cause. Participants in the trial had a higher age at death than non-participants | Gut | 2010 | X |  | X |  |  |  |  |  |
| Wilding | Do reasoned action approach variables mediate relationships between demographics and cervical cancer screening intentions or behaviour? An online study of women from the UK | Younger women report higher intentions to participate. White women report higher intention to participate. Women from less deprived areas report high intention to participate | Social Science & Medicine | 2022 | X |  | X | X |  |  |  |  |
| Williams | Ethnic differences in barriers to symptomatic presentation in primary care: A survey of women in England | Ethnic minority women reported a higher number of barrier and emotional barriers were prominent | Psycho-Oncology | 2019 |  |  |  | X |  |  |  |  |
| Wittmann | Comparison of patients' needs and doctors' perceptions of information requirements related to a diagnosis of oesophageal or gastric cancer | Increasing age negatively associated with internet access and so ability to access information. No difference between male and females with regards to information given. SES not related to patient information need but increasing deprivation associated with reduced access to internet and so information | European Journal of Cancer Care | 2011 | X |  | X |  |  |  |  | X |
| Woodhead | Cervical and breast cancer screening uptake among women with serious mental illness: a data linkage study | Women with severe mental illness are less likely to receive breast and cervical cancer screening than comparable women without mental illness | BMC Cancer | 2016 |  | X |  |  |  |  |  |  |
| *Woods* | *Are deprivation-specific cancer survival patterns similar according to individual-based and area-based measures? A cohort study of patients diagnosed with five malignancies in England and Wales, 2008-2016* | *Individual vs area-level socioeconomic associations are different* | *BMJ Open* | *2022* |  |  | *X* |  |  |  |  |  |
| Woods | Are socio-economic inequalities in breast cancer survival explained by peri-diagnostic factors? | Survival was lower amongst women living in deprived areas but this was not explained by peri-diagnostic factors | BMC Cancer | 2021 |  |  | X |  |  |  |  |  |
| Wright | Identifying Social Distress: A Cross-Sectional Survey of Social Outcomes 12 to 36 Months After Colorectal Cancer Diagnosis | People under the age of 55 were more likely to experience social distress after cancer. People living in more deprived areas were more likely to experience social distress after cancer. People from non-White ethnic groups were more likely to experience social distress after cancer | Journal of Clinical Oncology | 2015 | X |  | X | X |  |  |  |  |
| Xie | A global assessment of the male predominance in oesophageal adenocarcinoma | There is a high sex difference in oesophageal adenocarcinoma in the UK | Oncotarget | 2016 |  |  |  |  |  |  |  | X |
| Zaakouk | Clinical, Histopathologic Features and Outcome of Breast Cancer in UK Women of Ethnic Origin | Non-White women more likely to present at a younger age, symptomatically, with unfavourable disease characteristics and have worse survival than White women | Asian Pacific Journal of Cancer Prevention | 2022 |  |  |  | X |  |  |  |  |
| Zeitler | Deprivation in relation to urgent suspicion of head and neck cancer referrals in Glasgow | Cancer diagnosis more common in referrals for people in the most deprived areas. Cancers were more common in women being urgently referred | Clinical Otolaryngology | 2018 |  |  | X |  |  |  |  | X |
